# Ion channel gene *GJB2* influences the intercellular communication by Up-regulating the SPP1 signaling pathway identified by the single-cell RNA sequencing in lung adenocarcinoma

**DOI:** 10.3389/fonc.2023.1146976

**Published:** 2023-04-28

**Authors:** Zuo Liu, Zengtuan Xiao, Xiaofei Wang, Lianmin Zhang, Zhenfa Zhang

**Affiliations:** Department of Lung Cancer Surgery, Tianjin Medical University Cancer Institute and Hospital, National Clinical Research Center for Cancer, Key Laboratory of Cancer Prevention and Therapy, Tianjin's Clinical Research Center for Cancer, Tianjin, China

**Keywords:** *GJB2*, SPP1, lung adenocarcinoma, immune infiltration, intercellular communication

## Abstract

**Objective:**

Firstly, observe the prognostic significance and the biological functional effects of gap junction protein beta 2 (*GJB2* or Cx26) in lung adenocarcinoma (LUAD). Subsequently, explore the role played by *GJB2* in intercellular communication by single-cell RNA sequencing.

**Method:**

We made a differential analysis of *GJB2* expression through public databases and investigated the clinical characteristics and prognostic significance. ESTIMATE analysis and Tumor Immune Estimation Resource (TIMER) database were utilized to illustrate the association of *GJB2* with immune infiltration and components of the tumor microenvironment. Gene Ontology (GO), Kyoto encyclopedia of genes and genomes (KEGG), and Gene set enrichment analysis (GSEA) were performed to study the biological function of *GJB2*. Cell-cell communication was analyzed using the CellChat R package through sc-RNA data.

**Results:**

*GJB2* has an outstanding prognosis value in LUAD and a close relationship was found between *GJB2* and immune infiltration in LUAD. *GJB2* could participate in several tumor biological processes, including extracellular matrix remodeling and upregulation of multiple cancer-related active pathways. *GJB2* related hub-genes influence intercellular communication through the SPP1 signaling pathway.

**Conclusion:**

Our study illustrates one mechanism by which *GJB2* exerts its cancer-specific relevant effects, that is, causing changes in intercellular communication through the SPP1 signaling pathway. Blockade of this pathway may limit the functional role of *GJB2* and provide us with promising new perceptions for LUAD treatment.

## Introduction

As is well-known, lung cancer is the most common and fatal tumor in the world (1). The same condition also occurs in China according to the latest national cancer report released by the National Cancer Center in 2022. This report demonstrated that there were about 820 thousand new cases of lung cancer and about 710 thousand deaths in 2020, accounting for about 23.8% of cancer deaths, and lung cancer is the primary cause of cancer-related deaths, both in men and women (2). Therefore, the five-year survival rate of lung cancer is still not ideal, only 15% (3). Lung adenocarcinoma (LUAD), as an important subtype of non-small cell lung cancer (NSCLC), has the highest incidence rate among NSCLC. The occurrence and progression of LUAD is a complicated multi-step process, which might strongly correlate with the anomalous expression of several genes. Consequently, the development of molecular mechanism of LUAD may identify more accurate targets and therapeutic strategies, which is necessary for LUAD diagnosis and treatment and enhance the prognosis (4, 5).

Gap junction protein beta 2 (*GJB2*), namely, connexin 26 (Cx26), is localized at chromosome 13q11-12, encoding a membership of the gap junction protein family. Because of the differences and similarities in the connexin sequences, this family could be defined into 5 connexin subfamilies (including α, β, γ, δ, and ϵ or GJA, GJB, GJC, GJD, and GJE). Gap junctions are the structures in membrane surface that contributes to the direct communication between cells. And it’s well known that collaborative communication is at the heart of multicellular life (6). Previous studies have demonstrated the prognostic effect of *GJB2* on lung cancer starting from the perspective of its ion channel action (7). In other oncological aspects, *GJB2* was also reported as an oncogene related to tumor growth and metastasis in colorectal cancer (8), esophageal cancer (9), and breast cancer (10). Hence, we propose the hypothesis that the tumor-promoting effect of *GJB2* may correlate with the function of intercellular communication. Subsequently, we conducted this study to detailed clarify the functional significance of *GJB2* and further investigated its possible impact on cell-cell chat by single-cell sequencing analysis in LUAD.

In this study, firstly, we validated the clinical significance and prognostic value of *GJB2* through the Cancer Genome Atlas (TCGA). Then we obtained the differentially expressed genes (DEGs) associated with *GJB2* by taking intersection with Gene Expression Omnibus (GEO) dataset and performed a multi-omics analysis and functional analysis on them. Finally, the variation in cellular communication caused by DEGs was explored through a GEO single-cell dataset.

## Materials and methods

### Data source

The RNA-seq data for 598 samples consisting of 539 tumor and 59 normal samples with clinical information was downloaded from TCGA (https://cancergenome.nih.gov/) and transcripts per million (TPM) normalized. GEO dataset GSE31210 contains 246 LUAD patients’ expression profiles and then takes intersections with the TCGA database to obtain *GJB2*-related DEGs. The GSE171145 dataset from GEO database collected 40,799 single cells from nine samples of eight LUAD patients and was utilized for analyzing the DEGs-related functions.

### Detecting the differential expression of *GJB2*

The differential expression level of *GJB2* in pan-cancer was downloaded from the TIMER (Tumor Immune Estimation Resource) (https://cistrome.shinyapps.io/timer/). The *GJB2* mRNA expression from the TCGA database in different clinical conditions was analyzed and then plotted by “limma”, “ggplot2”, and “ggpubr” packages. The CPTAC (Clinical Proteomic Tumor Analysis Consortium) database was utilized to explore the protein expression of GJB2 in LUAD. The Human Protein Atlas (HPA) (https://www.proteinatlas.org/) was utilized to show the immunohistochemistry (IHC) of *GJB2* in LUAD and nonmalignant samples (11).

Comparison of *GJB2* expression in normal and tumor tissues by acquiring data from TCGA and Genotype Tissue Expression project (GTEx) (12) which catalogued gene expression from healthy individuals. To address the batch effect in different samples and data processing from different sources, we referred to the processed data from Wang Q et al. which was successfully corrected for batch effects for TCGA and GTEx (13). The relevant data could be available on Figshare (https://doi.org/10.6084/m9.figshare.5330593).

### Clinical characteristics of *GJB2* expression

The heatmap and correlation analyses between *GJB2* expression and underlying clinical parameters were investigated by “limma”, “ggpubr” and “ComplexHeatmap” R language packages. Nomograms and calibration curves were, respectively, drawn by “survival”, “survminer”, “regplot” and “rms” packages. Univariate and multivariate COX regression analyses were conducted separately, then corresponding results were visualized by forest plots.

### Identification of DEGs

All data were processed by R software. “Limma” package was utilized for identifying DEGs between the *GJB2* high-expression group and *GJB2* low-expression group in the TCGA and GEO datasets. An adjust P value < 0.05 and the absolute log2 fold change (logFC) > 1 were defined as the screening criteria for DEGs. Heatmaps and volcanic maps of DEGs were constructed and the overlapping DEGs between GSE31210 and TCGA were displayed through the VennDiagram.

### PPI network and the top 11 hub-genes

The online tool Search Tool for the Retrieval of Interacting Genes (STRING, http://string-db.org) (14) was utilized to get interactive relationships of the overlapping DEGs. A Confidence score ≥ 0.4 was considered significant. The results of the analysis were imported into Cytoscape 3.9.1 (http://www.cytoscape.org/) (15) to establish and visualize a PPI (Protein-protein interaction) network model. Subsequently, the plug-in app cytoHubba (16) was used to select the top 11 hub-genes from the PPI network according to the node degree.

### Functional analysis of *GJB2*-related genes

Gene ontology (GO) and Kyoto Encyclopedia of Genes and Genomes (KEGG) pathway enrichment analyses were performed based on the *GJB2*-related DEGs by “clusterProfiler” package of R language, and relevant result visualization was conducted by “enrichplot” package of R software.

### GeneMANIA

GeneMANIA (http://genemania.org/) is an easy to use web-portal for studying protein-protein interactions according to gene functions (17). The GJB2 protein association network was constructed from the GeneMANIA tool based on physical interactions, co-expression, predicted, co-localization, pathway, and genetic interactions.

### Gene set enrichment analysis

To further investigate the potential functional effects of *GJB2*, we divided the LUAD individuals from the TCGA database into two groups according to the median expression level of *GJB2* and conducted GSEA software version 4.2.2 (www.gsea-msigdb.org/gsea/index.jsp) (18) to explore whether genes that were differentially regulated between the two groups were enriched in cancer-related biological pathways. The annotated gene sets, c2.cp.kegg.v2022.1.Hs.symbols.gmt and h.all.v2022.1.Hs.symbols.gmt, were selected as the reference gene set. FDR (q value) < 0.05 and P value < 0.05 were considered statistically significant. Finally, the log fold change (FC) values calculated by the software were imported into the R software and visualization analysis of the data was conducted by “clusterProfiler”, “enrichplot” packages.

### Correlation between *GJB2* and tumor immune infiltrating cells

To clarify the underlying immunomodulatory mechanism of *GJB2*, we evaluated the correlation between *GJB2* and multiple immune infiltrating cells and immune checkpoints in TCGA-LUAD samples, which were calculated by the CIBERSORT algorithm and then visualized using R language packages. In addition, the relationship between *GJB2* copy number variation (CNV) and various immune infiltrates was explored by somatic copy number alteration (SCNA) mode from the TIMER database.

### The sc-RNA seq data analysis

Using the Seurat package in R software, Seurat objects were created for each sample with the cell-by-gene count matrix (min.cells =3, min.features =250). Then we reserved the cells of nFeature_RNA>50 and mitochondrial gene percentage<10%. After normalization, the top 1500 genes, selected as the top variable features, were used as principal component analysis (PCA) by using the FindVariableFeatures of the Seurat package. Significant principal components (PCs) were identified using the pcaJackStraw function. Then, the top 15 PCs were utilized to the “TSNE (t-distributed stochastic neighbor embedding)” dimensionality reduction. The resolution was set =0.5 and the cells were clustered by the FindClusters function. Finally, the specific cluster’s marker genes were identified by FindAllMarkers function. Cellular clusters were annotated using the Cell-Marker 2.0 dataset (19) to identify the cell types to which multiple clusters belong.

### Intercellular communication analysis

Cell-cell communication was determined using the CellChat R package that contains ligand-receptor interaction databases for human and mouse which can describe the cell-cell chat networks through sc-RNA data to compute intercellular communication within the identified cell subtypes (20). Then, the relevant visualization function in this R package was utilized to show the intercellular communication networks from the target cell cluster to different cell clusters.

### Statistical analysis

Kaplan-Meier Plotters and corresponding log-rank tests were used to analyze the survival status. The correlation of *GJB2* with various immune cell infiltration was calculated by Spearman’s test. A positive correlation was defined as an R-value >0.1, while a negative regulatory relationship was defined as an R-value <–0.1. P value <0.05 was thought statistically significant (*P<0.05, **P<0.01, ***P<0.001).

## Results

### Multi-perspective analysis of *GJB2* expression

**Figure 1:**
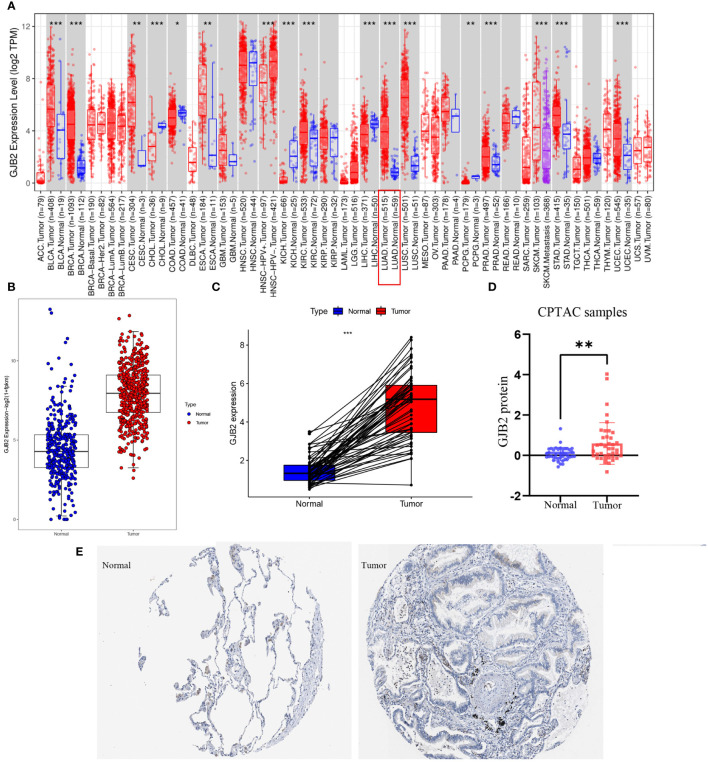
*GJB2* was upregulated in LUAD. **(A)** *GJB2* expression levels in diverse kinds of tumors. Data was extracted from the TCGA database by TIMER. **(B)** *GJB2* expression in LUAD tissues compared to that in nonmalignant lung tissues. **(C)** *GJB2* expression pre-disease and post-disease in the same patient. **(D)** Differential expression of GJB2 protein between LUAD and nonmalignant lung tissues in the CPTAC data resource. **(E)** *GJB2* immunochemistry in LUAD and normal tissue using data from HPA. *P<0.05, **P<0.01, ***P<0.001.

First of all, we evaluated *GJB2* expression in different types of tumors by the TIMER database and concluded that the high expression of *GJB2* was associated with a variety of cancers, and LUAD is one of the affected tumors (**Figure 1A**). Subsequently, by analyzing the processed data corrected for batch effects, we found that the expression of *GJB2* in LUAD tumor tissues (483 samples in TCGA) was higher than that in nonmalignant lung tissues (313 samples in GTEx, 59 samples in TCGA) (**Figure 1B**). In addition, for the same individual, the expression of *GJB2* in the normal samples (pre-disease) and LUAD tissues (post-disease) was different. The *GJB2* in the tumor condition was elevated meaningfully, indicating that the content of *GJB2* was upregulated with the LUAD progressing (P < 0.001) (**Figure 1C**). Next, the difference in GJB2 protein expression between the two groups was explored through the CPTAC database and LUAD group showed a relatively higher-level contrast to the normal group (P =0.004, **Figure 1D**). Eventually, the IHC result was also obtained from HPA database (**Figure 1E**).

### The *GJB2* mRNA expression and clinical characteristics of LUAD

**Figure 2:**
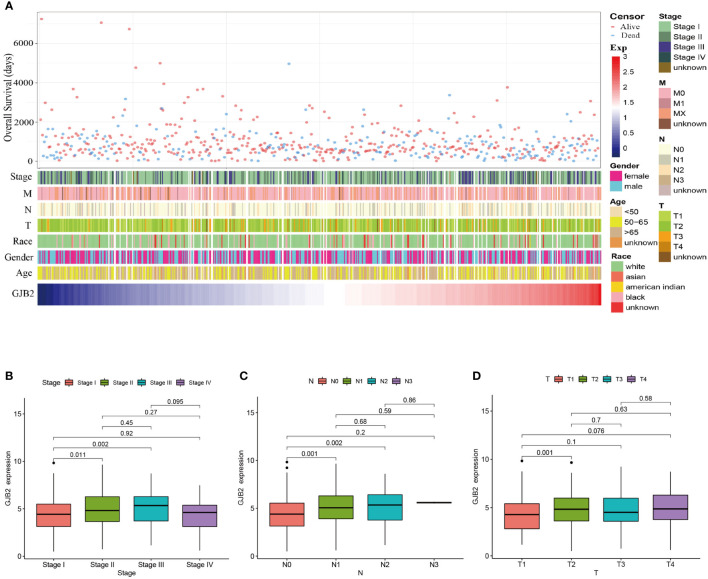
Association between *GJB2* mRNA expression and clinical characteristics of LUAD. **(A)** The landscape of *GJB2*-related clinical features of LUAD in the TCGA database. **(B–D)** Comparison of *GJB2* mRNA expression based on patient’s individual cancer, nodal metastasis, and tumor stage.

Patients with varying expression levels of *GJB2* showed distinct patterns of clinical characteristics. Increasing *GJB2* expression, age distribution, gender, race, T stage, N stage, M stage, and tumor TNM stage showed asymmetric distribution in the TCGA database, while patients’ OS displayed a declining tendency (**Figure 2A**). Comparative analysis was also performed on different groups of these samples. Results showed that *GJB2* was most highly enriched in stage III LUAD and there was a distinct expression difference between stage I and stage II(P=0.011) and between stage I and stage III (P=0.002, **Figure 2B**). Also, increased in tumor N stage, the expression of *GJB2* gene tended to rise and a significant difference was observed between N0 and N1(P=0.014), between N0 and N2 (P=0.016, **Figure 2C**). Moreover, when the tumor progressed from T1 to T2, the *GJB2* gene expression was also elevated clearly (P=0.001, **Figure 2D**). Thus, the expression of *GJB2* was independent of factors such as age, gender and race, but it shows an upward trend as the disease progresses.

### Prognostic value of *GJB2* in LUAD

**Figure 3:**
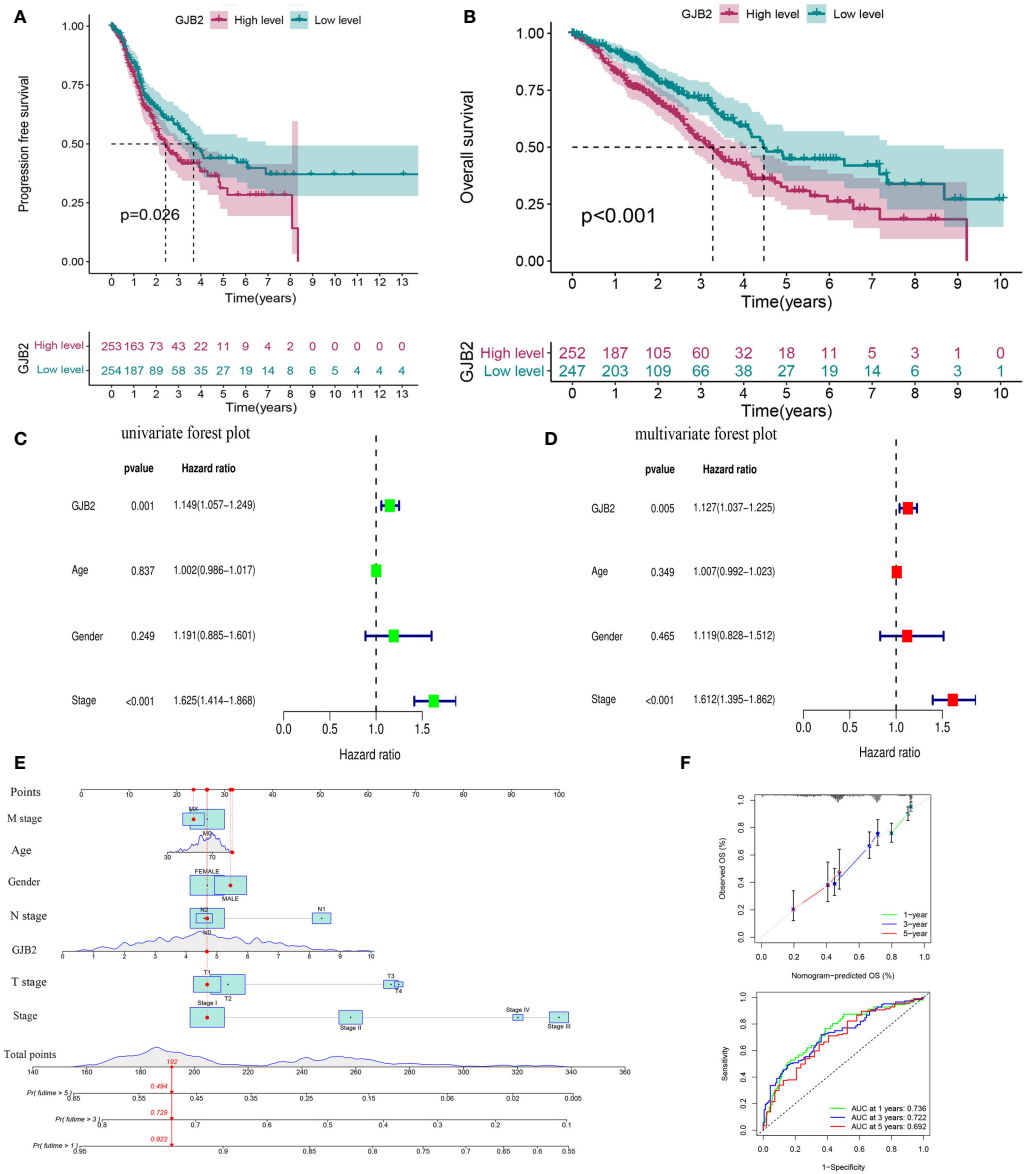
Prognostic value of *GJB2* in LUAD. **(A)** Progression-free survival curve based on TCGA-LUAD. **(B)** Overall survival curve based on TCGA-LUAD. **(C)** Univariate Cox regression analysis of prognosis-related risk factors. **(D)** Multivariate Cox regression analysis of prognosis-related risk factors. **(E)** A nomogram for predicting survival of LUAD. **(F)** A calibration curve and ROC curve for the nomogram model.

The up-regulated *GJB2* expression was associated with worse prognosis in LUAD, while patients with down-regulated *GJB2* expression showed a better survival both in PFS (progression-free survival) and OS (P =0.026, P<0.001, **Figures 3A, B**). The median OS for the individuals in the high *GJB2* expression group was 3.28 years (range: 2.74–4.09 years), and the median OS for the low *GJB2* expression group was 4.47 years (range: 4.10–8.68 years). Therefore, we thought that the expression of *GJB2* was closely associated with the progression of LUAD. And a related prognostic analysis was performed.

Univariate and multivariate Cox analyses were performed to analyze the clinical characteristics of 512 samples with complete clinical information from the TCGA-LUAD dataset. In the univariate Cox analysis, the TNM stage (HR = 1.625, P<0.001, 95% CI: 1.414–1.868) and *GJB2* expression (HR = 1.149, P =0.001, 95% CI: 1.057–1.249) were significant prognostic factors for LUAD (**Figure 3C**). Then we conducted a multivariate Cox analysis and concluded that TNM stage (HR = 1.612, 95% CI: 1.395–1.862, P <0.001) and *GJB2* expression (HR = 1.127, 95% CI: 1.037–1.225, P =0.005) were still the independent prognostic factors for LUAD (**Figure 3D**). Subsequently, a prognostic nomogram including clinical characteristics parameters and *GJB2* expression was constructed to predict 1-, 3-, and 5-year overall survival according to the stepwise COX regression model (**Figure 3E**). The C-index of this prognostic nomogram was 0.685. The ROC curves of the model was depicted and the AUC values were 0.736, 0.722 and 0.692 at 1, 3 and 5 years, respectively. Calibration plots showed that the nomogram performed a good fit for predicting OS for LUAD patients (**Figure 3F**).

### Screening of the *GJB2*-related DEGs and construction of PPI network

**Figure 4:**
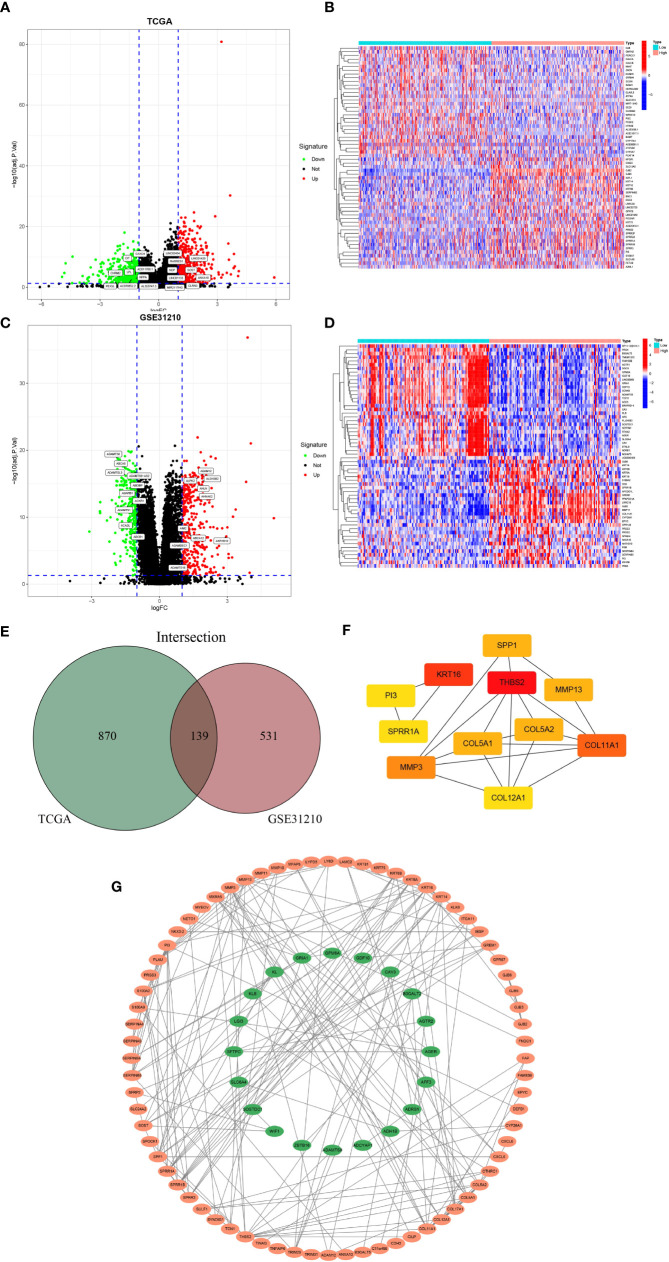
**(A)** Volcanic map of 1009 DEGs in TCGA and related representative genes. Red: up-regulation; green: downregulation; black: normally expressed mRNAs. **(B)** Heatmap of representative top 60 genes based on high and low *GJB2* expression group in TCGA. **(C)** Volcanic map of 670 DEGs in GSE31210 dataset and related representative genes. **(D)** Heatmap of representative top 60 genes based on high and low *GJB2* expression group in GSE31210 dataset. **(E)**Venn diagram shows the intersecting DEGs from GEO and TCGA. **(F)** Top 11 genes with high node degree in cytoscape. **(G)** Visualized PPI analysis of DEGs based on cytoscape.

We first screened out *GJB2*-related DEGs in the TCGA-LUAD database with the filtering criteria of |log_2_FC| > 1 and adj. p-value < 0.05. As a result, 1009 DEGs were extracted from the TCGA-LUAD dataset (**Figure 4A**). The top 30 genes with positive or negative correlation with *GJB2* were displayed in the heatmap (**Figure 4B**). To further obtain accurate *GJB2*-associated differential genes, gene expression microarrays regarding LUAD were searched in the GEO database. And the expression profile chip data GSE31210 were found which contained 246 LUAD samples. Volcanic map and heatmap showing representative DEGs associated with *GJB2* in **Figures 4C, D**. The results of DEGs from the TCGA and GEO databases are summarized in **Supplementary Table 1**. The Venn package was utilized to identify the intersecting DEGs from both databases, and generate the Venn map (**Figure 4E**). Eventually, 139 *GJB2*-related DEGs with high reliability were obtained which were designated for subsequent analyses. **Figure 4G** illustrates the protein-protein interaction (PPI) network among 139 genes, where the orange pattern in the outer circle represents a positive correlation with *GJB2* expression, in contrast to the green pattern in the inner circle, which represents a negative regulatory relationship. And the top 11 hub-genes from the PPI network were selected with high node degree with the help of the plug-in app cytoHubba in Cytoscape software (**Figure 4F**).

### Functional analysis of *GJB2*-related DEGs

**Figure 5:**
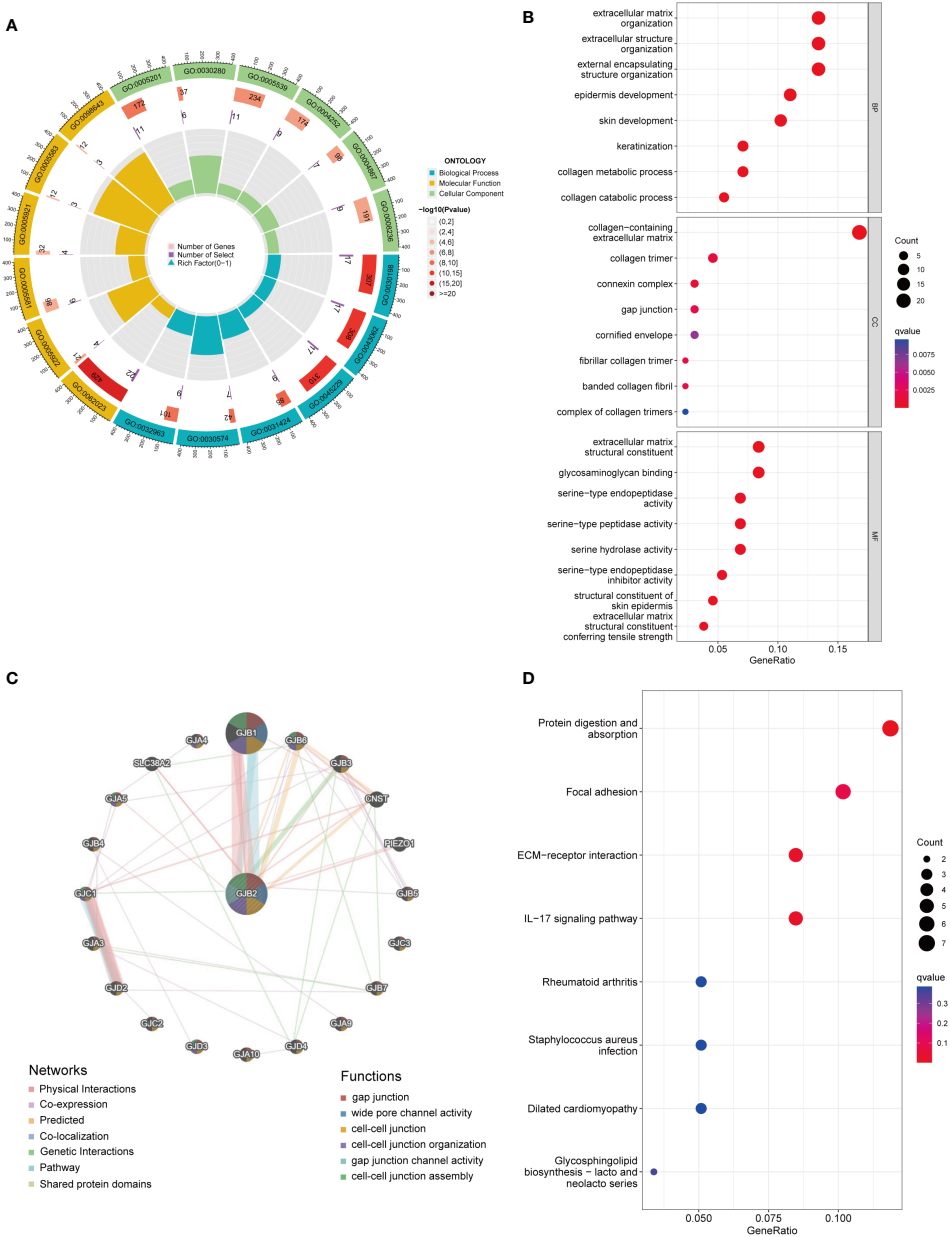
**(A)** GO circle map of *GJB2*-associated DEGs. **(B)** Enrichment analysis of GO terms for *GJB2*-related DEGs. **(C)** Interaction networks between *GJB2* and its interactive genes using GeneMANIA. **(D)** Enrichment analysis of KEGG terms for *GJB2*-related DEGs.

To predict the potential biological function and corresponding pathways of these significant DEGs, Gene Ontology (GO) and Kyoto Encyclopedia of Genes and Genomes (KEGG) pathway analyses were performed. With the filtering criteria of p.adjust < 0.05 and q value < 0.05, *GJB2*-related DEGs were mainly enriched in 61 biological processes (GO-BP), 17 cell components (GO-CC), 35 molecular functions (GO-MF) and 3 KEGG (**Supplementary Table 2**). Meanwhile, we combined the results from GeneMANIA, which was more helpful to understand protein-protein interactions and *GJB2* gene family functions (**Figure 5C**). The results of GO term enrichment analysis varied from GO classification. As for the biological processes, we discovered that the *GJB2*-associated genes were mainly enriched in extracellular matrix organization, extracellular structure organization, and external encapsulating structure organization (**Figures 5A, B**). Additionally, *GJB2* was enriched in the collagen−containing extracellular matrix, collagen trimer, and connexin complex (**Figure 5B**). Also, we found that *GJB2* was associated with the molecular functions of extracellular matrix structural constituents and extracellular matrix structural constituents conferring tensile strength (**Figure 5B**). KEGG pathway analysis also revealed the functional enrichment in ECM-receptor interaction, focal adhesion, and IL-17 signaling pathway (**Figure 5D**).

### Gene set enrichment analysis

**Figure 6:**
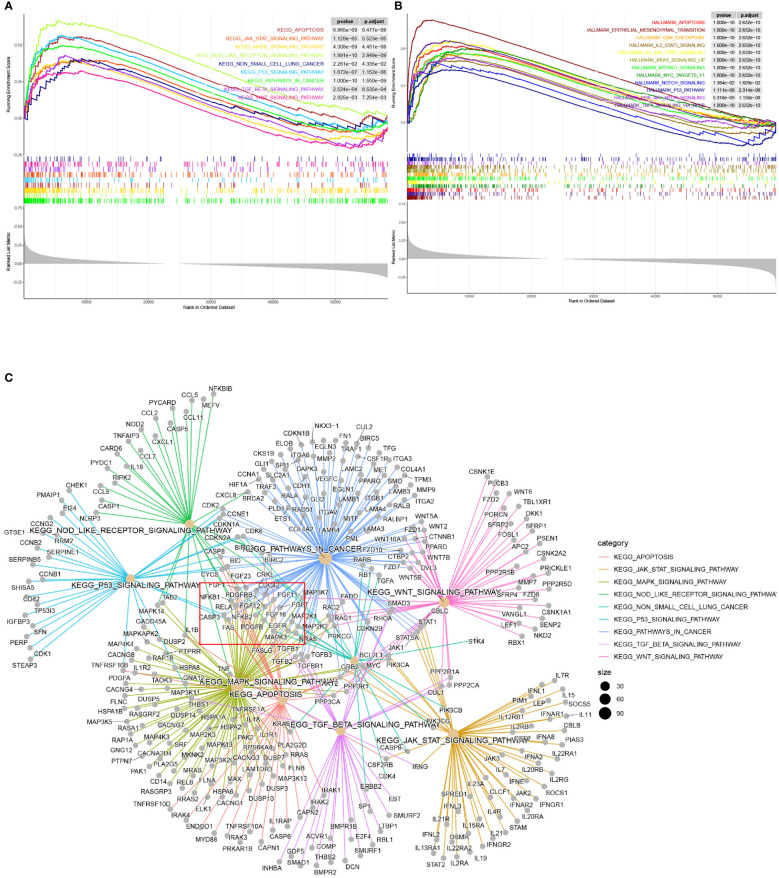
**(A)** Gene Set Enriched Analysis based on kegg.v2022.1 gene sets and significant LUAD-related enriched pathways. **(B)** Gene Set Enriched Analysis based on hallmark gene sets and significant LUAD-related enriched pathways. **(C)** The association network diagram of LUAD-related signaling pathways.

Based on the target sets c2.cp.kegg.v2022.1, a total of 98 gene sets were found with the screening criteria, both p* *value and q value < 0.05. Among them, several important LUAD-related pathways could be observed significantly enriched, including pathways in cancer (P<0.001), nod-like receptor signaling pathway (P<0.001), MAPK signaling pathway (P<0.001), apoptosis (P<0.001), P53 signaling pathway (P<0.001), JAK-STAT signaling pathway (P<0.001), TGF-Beta signaling pathway (P<0.001), WNT signaling pathway (P=0.003), non-small cell lung cancer pathway (P=0.023)(**Figure 6A**). Detailed enrichment analysis information was displayed in **Supplementary Table 3**. Furthermore, through the association network diagram composed by the enriched functional pathways, we found that some genes can be involved in multiple signaling pathways (**Figure 6C**).

Based on the target set hallmark, a total of 45 gene sets were found with the screening criteria, both p* *value and q value <0.05, including gene sets associated with apoptosis, epithelial mesenchymal transition, G2M checkpoint, IL2-STAT5 signaling, IL6-JAK-STAT3 signaling, KRAS signaling up, m-TORC1 signaling, MYC targets V1, NOTCH signaling, P53 pathway, PI3K-AKT-mTOR signaling, TNFA signaling *via* NFKB (**Figure 6B**). Detailed enrichment analysis results are shown in **Supplementary Table 3**. And the ridge plot showed the distribution and overlap of core genes of the first 25 enriched gene sets in the GSEA analysis (**Supplementary Figure S1**).

### Correlation analyses of *GJB2* with immune infiltration and immune checkpoints

**Figure 7:**
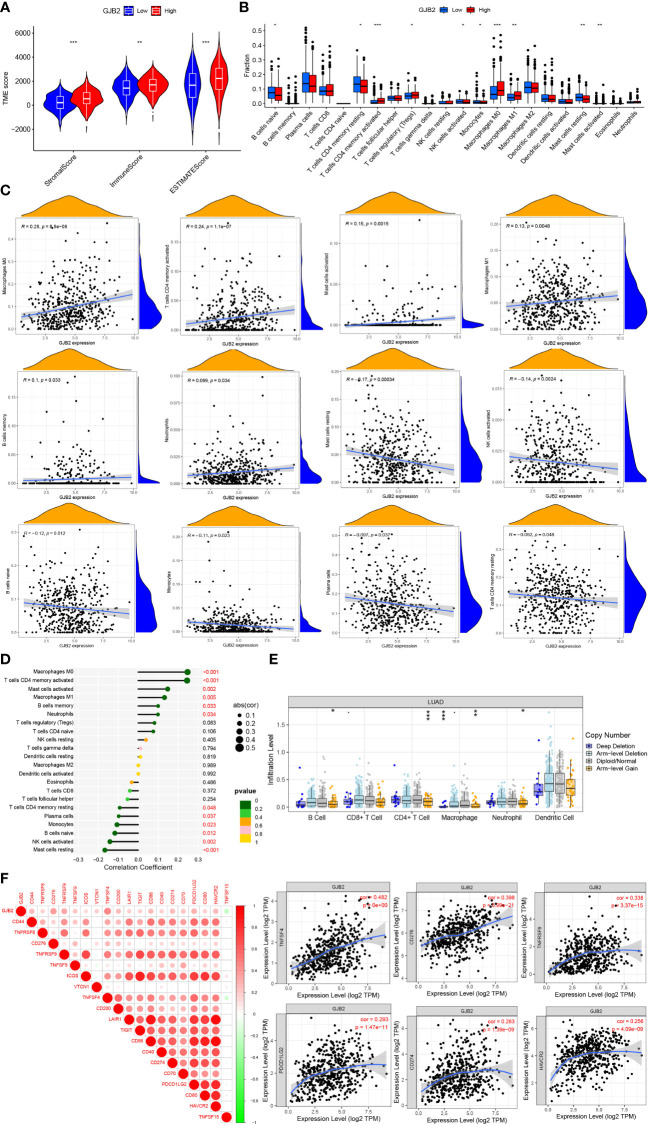
**(A)** Immune-score difference between *GJB2* high-expression group and low-expression group. **(B)** Changes of 22 immune cell subtypes between high and low *GJB2* expression groups in LUAD tumor samples. **(C, D)** Correlation between *GJB2* and immune infiltrating cells in LUAD. **(E)** *GJB2* CNV affects the infiltrating levels of B cells, CD4+ T cells, macrophages, and neotrophils in LUAD. **(F)** Correlations between *GJB2* and various immune checkpoints. *P<0.05, **P<0.01, ***P<0.001.

We also attempted to investigate whether *GJB2* was associated with immune infiltration and immune checkpoint expression. Firstly, by the ESTIMATE analysis, we found there was a distinct immune-score difference between *GJB2* high-expression group and *GJB2* low-expression group (**Figure 7A**). To further explore the *GJB2*-associated immune infiltration level, we elucidated the correlation between *GJB2* and different immune infiltrates (**Figure 7B**). Specifically, the high *GJB2* expression correlated with a unique infiltrating degree of the immune cell populations. For example, M0 macrophages, cluster of differentiation (CD)4 memory-activated T cells, activated mast cells, and M1 macrophages were positively correlated with *GJB2* (R>0.1, P<0.05) while resting mast cells, activated NK cells, naive B cells, and monocytes were negatively correlated with *GJB2* (R<-0.1, P<0.05) (**Figures 7C, D**). In addition, we compared the immune cell infiltration levels in LUAD with different somatic copy number alterations in *GJB2*. Copy number in arm-level gain spanning the *GJB2* gene locus was associated with decreased immune cell infiltration in CD4+ T cells or macrophages. And copy number deep deletion in *GJB2* was also associated with decreased immune infiltrates in macrophages (**Figure 7E**). Finally, the results of correlation analysis of immune checkpoints from the TIMER database indicated that *GJB2* was positively correlated with TNFSF4, CD276, TNFRSF9, PDCD1LG2, CD274, and HAVCR2 (R>0.25 P<0.01; **Figure 7F**). In conclusion, our research results revealed the crucial significance of *GJB2* on immune infiltration levels and correlations between immune checkpoints in LUAD.

### Cellular composition in single cell RNA-seq dataset

**Figure 8:**
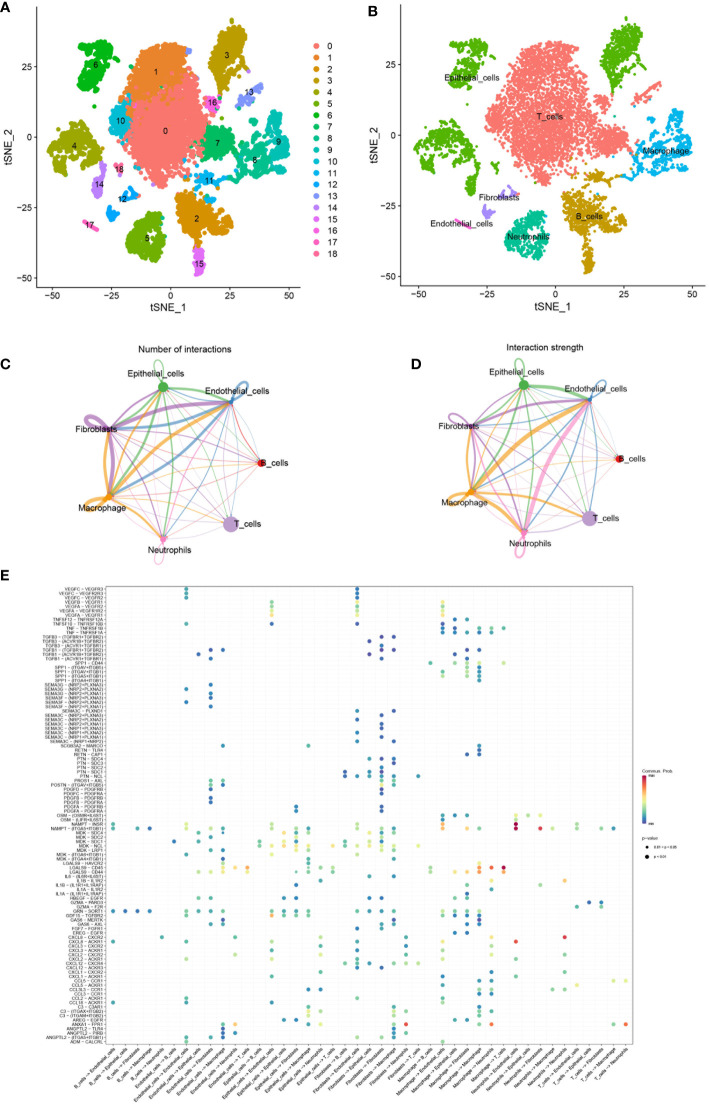
**(A)** TSNE plots of all cells used in this study which are annotated according to cell clusters. **(B)** TSNE plots of scRNA-seq data for seven cell types. **(C, D)** Interaction network of seven cell types constructed by CellPhoneDB, where the thicker line indicates more interactions with other cell types. The arrow emitter is the ligand while the recipient is the receptor. **(E)** The community probability of all the ligand-receptor pairs during interactions between different cell types in this single-cell dataset.

By the *GJB2*-related DEGs functional analysis, we identified the important role of *GJB2* in the extracellular matrix (ECM) activity and ECM−receptor interaction. Analysis of the tumor microenvironment also suggested an intrinsic rich infiltration of immune cells. Because of the unique role of the *GJB2* itself, we attempted to explain these biological processes from the perspective of intercellular communication and elucidate what kind of role the ion channel gene *GJB2* plays in this process. Therefore, a scRNA-seq data analysis was performed. After searching for the GEO database, GSE171145 which collected 40,799 single cells from nine samples of eight LUAD patients caught our attention. Finally, single-cell data for 11,759 cells of nine LUAD samples were included after quality filtering (**Supplementary Figure 2**). Unsupervised clustering analysis and the TSNE method were performed for visualizing nineteen cell clusters according to the most important differentially expressed genes in different cell clusters (**Figure 8A**). Detailed information of the marker genes for each cluster is shown in **Supplementary Figures 3A, B**. Finally, they were annotated with seven common major cell types, including T cells, B cells, macrophages, epithelial cells, neotrophils, fibroblasts, and endothelial cells in nine samples from eight LUAD patients (**Figure 8B**). We then fully evaluated the significantly different expression for the mean RNA expression of *GJB2*-related DEGs in the LUAD samples by using the AverageExpression function of Seurat. And the mean RNA expression of the hub-genes in cytoscape networks was shown by violin plot and scatter plot with significantly different percentages of major cell types. But due to sample reasons, the expression of MMP3 and SPRR1A in the hub-genes was not detected in the dataset (**Supplementary Figure 4**).

### Global comparative analysis of intercellular communication in LUAD

In order to study the interactions of multiple kinds of cells in the LUAD TME, the strength and number of interactions between ligand-receptor pairs among 7 cell types in our dataset were investigated by cellphoneDB. Extensive and active intercellular information exchange can be observed. The number of different types of intercellular interactions is shown in **Figure 8C**. The global interaction network in the selected sc-RNA seq dataset among the 7 cell types is depicted in **Figure 8D**, where the thicker line implicates more interactions with other cell types. It was also observed that macrophages, epithelial cells, fibroblast, and endothelial cells were more active in intercellular interactions among clusters. Furthermore, the bubble map in **Figure 8E** indicated the community probability of all the ligand-receptor pairs that exert an important role during interactions between different cell types in LUAD TME.

### Intercellular communication influenced by hub-genes

**Figure 9:**
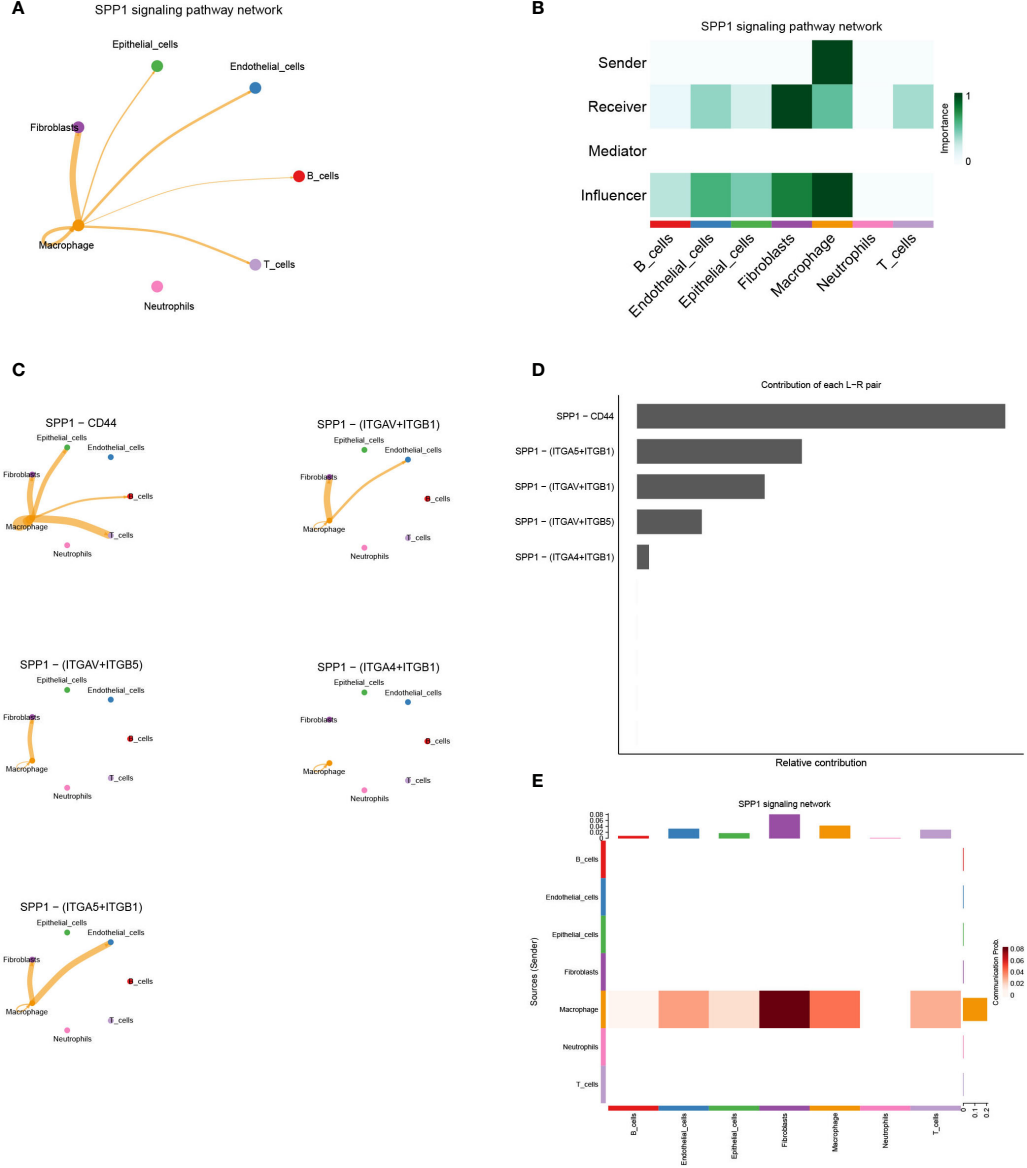
**(A)** Cell–Cell communications in SPP1 signaling pathway. **(B)** The role of each cell type in intercellular communication exchange. **(C)** Five types of ligand-receptor pairs in the SPP1 signaling pathway. **(D)** Contribution of each ligand−receptor pair in the SPP1 signaling pathway. **(E)** The intercellular communication probability between different cell types in the SPP1 signaling network.

By comparing the TCGA database with the GEO database, we obtained the hub-genes that were influenced by *GJB2* and detected their expression in single-cell datasets. Among many ligand-receptor pairs mediating the exchange of information between cells, our research found that the hub-genes could be involved in cellular ligand-receptor pair information communication mediated by the SPP1 signaling pathway (**Figure 9A**). In the SPP1 signaling pathway network, macrophage act as senders, and fibroblasts, epithelial cells, endothelial cells, B cells, T cells, and macrophage act as receivers thus enabling intercellular communication (**Figure 9B**). And in this cell-chat communication, the ligand SPP1 interacts between cells through five kinds of receptors on different types of cell membranes (**Figure 9C**). Among them, the SPP1-CD44 axis plays an important role in this signaling pathway and other interacting ligand-receptor pairs include SPP1-αvβ1 integrin, SPP1-αvβ5 integrin (**Figure 9D**). **Figure 9E** also indicated the intercellular communication probability, which means that a higher frequency of information exchange between macrophages and fibroblasts is often achieved through the SPP1 signaling pathway. And the expression of ligand-receptor genes associated with this signaling pathway in different cell types is shown in **Supplementary Figure 4**.

### SPP1 signaling pathway in the *GJB2* differentially expressed groups

**Figure 10:**
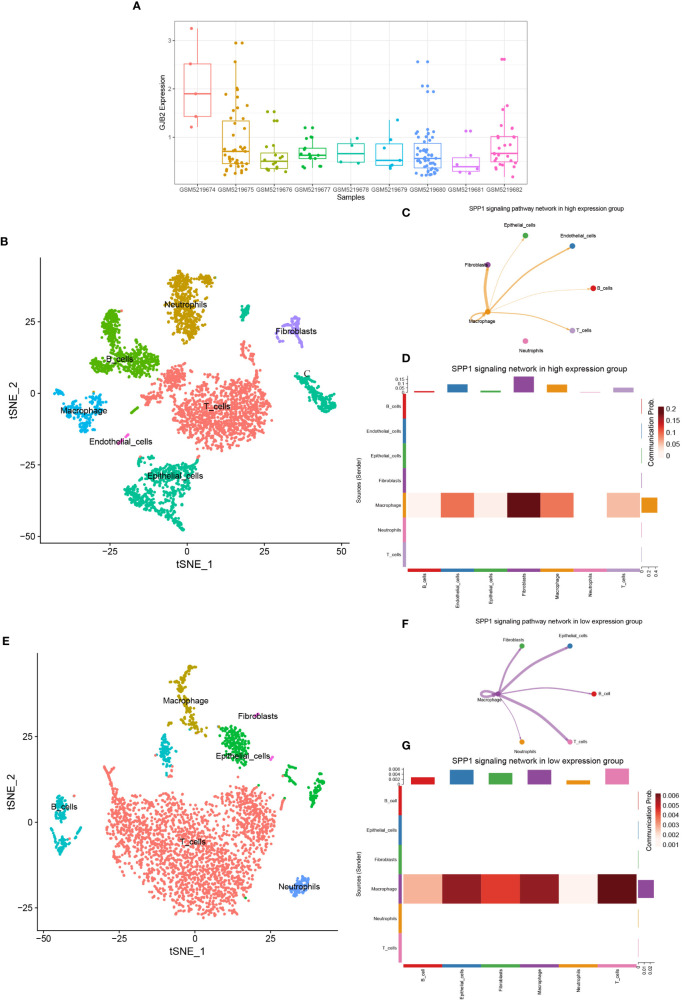
**(A)** GJB2 expression in each sample in the single-cell dataset. **(B)** TSNE plots of GJB2-high expression scRNA-seq data. **(C)** SPP1 signaling pathway network in high expression group. **(D)** The intercellular communication probability between different cell types in the SPP1 signaling network in high expression group. **(E)** TSNE plots of GJB2-low expression scRNA-seq data. **(F)** SPP1 signaling pathway network in low expression group. **(G)** The intercellular communication probability between different cell types in the SPP1 signaling network in low expression group.

By analyzing the GSE171145 dataset, we obtained the expression of *GJB2* in each sample, and then selected three samples (GSM5219675, GSM5219680, GSM5219682) with high expression of *GJB2* as the high expression group and three samples (GSM5219678, GSM5219679, GSM5219682) with low expression as the low expression group (**Figure 10A**). Using the same method, the cells in the two groups were clustered into several cell types. The cells in the high expression group were divided into the same 7 types, but the cells in the low expression group could only be divided into 6 types due to the low distribution of endothelial cells (**Figures 10B, E**). The expression distribution of *GJB2* and related hub-genes are shown in **Supplementary Figure 5**. Most of these genes are expressed in cancer-associated fibroblasts, and SPP1 is mainly expressed in macrophages. Upregulation of these genes mainly reinforces the cell-communication between macrophages and fibroblasts. **Figures 10C, F** shows the SPP1 signaling pathway in the two groups, respectively. And **Figures 10D, G** detailly illustrates the communication probability between different cells in this signaling pathway, respectively. From this we can conclude that the high expression group has a stronger communication probability than the low expression group in this signaling pathway.

## Discussion

Based on our bioinformatics analyses, we found that *GJB2* expression level was remarkably elevated in LUAD tissues, both in mRNA and protein expression level. And the high expression in post-disease compared to pre-disease indicated that *GJB2* was significantly associated with the onset and development of LUAD. Subsequently, the correlation of *GJB2* with clinical characteristics in the TCGA-LUAD database implied that *GJB2* expression was positively related to tumor size, node metastasis condition, and clinical stage. Multivariate Cox analysis revealed that the expression of *GJB2* was an independent prognosis factor for LUAD. In summary, supplemented by the Kaplan-Meier Plotter results, our analysis demonstrated that *GJB2* could act as a prognostic biomarker and exert a role in prospective prediction for LUAD. However, further studies should be performed to elucidate the possible biological functions of *GJB2* in LUAD.

By analyzing the expression of *GJB2*-associated genes in the TCGA database and GEO database, we obtained the *GJB2*-associated DEGs. GO functional analyses of these DEGs showed that ion channel gene *GJB2* could exert a vital role in migration-related biofunctions like extracellular matrix organization, cell−matrix adhesion, cell−substrate junction, focal adhesion, collagen trimer, integrin binding, and so on. On the one hand, collagen and fibronectin are significant ingredients of the ECM needed to support cell motility. On the other hand, a related study showed connexin expression had also been related to elevated migration and invasiveness of cancer cells (21). Moreover, in our study, increasing the *GJB2* expression level, the lymph node stage gradually deteriorated (**Figure 2C**), which was consistent with the function of *GJB2*. Most of the current research, including several cancers, such as skin (22), prostate (23), and colorectal carcinoma (8), suggested the function of Cx26 or Cx43 being linked to enhancing the motility and aggressiveness of cancer cells. Polusani, S. R et. also explored the underlying mechanism of gap junction proteins in metastasis or other tumor progressions in Hela cells (24).

GSEA and KEGG functional analyses suggested high *GJB2* expression was closely correlated with several cancer-related signaling pathways. For instance, the dysregulation of apoptosis signaling pathways predicts the occurrence and progression of malignancies, which also contributes to therapeutic drug resistance and immune escape (25). The JAK/STAT signaling pathway could be activated by a variety of cytokines (26) and the high expression of JAK2 modules could result in the proliferation, invasion, and migration of LUAD cells (27). Nod-like receptor (NLR) signaling pathway plays an important role in regulating cytokine production, aberrant activation of NLRs could be observed in diverse malignancies, leading to the tissue microenvironment imbalance and elevating the neoplastic risk (28). In addition, by analyzing the correlation network plot of enriched genes with their functional/pathway gene sets, we found that some genes can be involved in multiple LUAD-related regulation pathways in *GJB2* high expression group, such as FGF family, PDGFB, PDGFRB, and so on. FGF family, namely, the fibroblast growth factor family, receiving signals from their receptors, FGFR1, FGFR2, FGFR3, or FGFR4, could take part in cellular bioprocesses, related metabolism and exert the role of signaling cascades in angiogenesis and immune evasion associated with oncology (29). Disordered FGF signals result in human diseases, such as breast cancer (30), colorectal cancer (31), and so on. In terms of the interaction between FGF and connexins, Kurt A. Schalpe et al. reported that FGF-1 transiently increased the membrane permeability through hemichannels composed of different connexins in Hela cells (32).

Based on the results of the ESTIMATE analysis, we discovered that high *GJB2* expression correlated with higher Immune Score and ESTIMATE Score, compared with the low expression group, which may demonstrate that *GJB2* could induce specific immune cell population infiltration. Through further analyses, we found that the expressions of macrophages M0 and M1 were important components of immune cell enrichment. Tumor-associated macrophages (TAMs), as the major infiltrating leukocytes of TME, exert a crucial role in the connection between inflammation and cancer (33). TAMs can kill tumor cells, promote tumor growth and angiogenesis, reshape tissue. Among them, M1 macrophages are thought to be tumor-killing macrophages, mainly exerting the role of anti-tumor and promoting-immune (34). Although an increased infiltration of M1 macrophages in the high GJB2 expressing group was observed, this is also a malignant feature of some tumors (35). Combing with the results of *GJB2* functional analysis, we found that *GJB2* exerted an important role in extracellular matrix remodeling. At the same time, macrophage polarization is also associated with the component changes in the TME, which includes conditions such as low pH, hypoxia, and ECM reshaping (36). Thus, the difference in macrophage between the two groups may correlate with the changes in* *extracellular* *matrix properties caused by *GJB2* expression. In addition, based on our bioinformatic analyses, the expression of *GJB2* always accompanies with some elevated expression of immune checkpoints, such as TNFSF4, CD276, TNFRSF9, PDCD1LG2, CD274, and HAVCR2. These modules, as immune checkpoint molecules, always played a costimulatory or coinhibitory dual role in the immune immunoregulatory system (37–39).

By the above analysis of bulk RNA-seq, we preliminarily demonstrate an important role for the ion channel gene *GJB2* in extracellular matrix remodeling and upregulation of cancer-related signaling pathways. Subsequently, we explored whether ion channel gene *GJB2* played a role in intercellular communication exchange at the single-cell level. After single-cell sample quality control and Cell-chat packages related analysis, we concluded that *GJB2* related hub-genes were involved in intercellular communication by influencing the SPP1 signaling pathway. SPP1, namely secreted phosphoprotein 1, was also known as osteopontin, which is a secreted chemokine-like glycophosphoprotein (40). SPP1 is a significant component of the extracellular matrix, secreted by many kinds of cell types including osteoclasts, fibroblasts, immune cells, and tumor cells (41). The interaction between SPP1 and CD44 played an important role in this signaling pathway. In this dataset, SPP1 is mainly expressed in macrophages and CD44 could be expressed in macrophages, fibroblasts, epithelial cells and T-cells. In the lung cancer study of Jane Zhou et al. they proved that exogenous activated SPP1 fragments can promote the migration and invasion of CL1-5 cells *in vitro*, and CD44 and αvβ3 integrin were important effectors in this process. The addition of separate αvβ3 integrin and CD44-specific antibodies greatly limited the migration and invasion ability of tumor cells (42). In colorectal cancer, related experiments also proved that the interaction between tumor-associated macrophages and CD44-positive cancer cells *via* SPP1-CD44 is important for colorectal cancer progression (43). Certainly, the altered intercellular communication induced by the upregulation of *GJB2* in tumor tissues may be reflected not only in the cell-chat between macrophage-epithelial cells through the reinforced SPP1-CD44 axis, thus promoting tumor progression, but also in the altered function of fibroblasts induced by the upregulation of *GJB2*-associated hub-genes. A newly published single-cell analysis in colon cancer liver metastases demonstrates the presence of a tight spatial proximity and cross-talk network between SPP1+ macrophages and fibroblasts, which contributes to reduced CD8+ T-cells function and upregulation of regulatory T cells forming a suppressive immune microenvironment (44). Therefore, from the results of the changes in intercellular communication in **Figure 10** and **Supplementary Figure 5**, it is also likely that *GJB2* and related genes contribute to tumor progression by upregulating the macrophage-fibroblast cross-talk and enhancing the immunosuppressive effects of fibroblasts.

The deficiency of this study should be mentioned that it is a bioinformatics analysis based on data mining and public databases, and relevant biological experiments should be further carried out to fully demonstrate the alteration of SPP1 signaling pathway among tumor cells, macrophages, and fibroblasts caused by the upregulation of *GJB2* in tumor tissues.

## Conclusion

In conclusion, based on our study, we confirmed that *GJB2* was up-regulated in LUAD, and its expression level was associated with clinical parameters and prognosis status of LUAD individuals. Additionally, bulk RNA-seq analysis elucidated that the expression of *GJB2* could associate with the status of immune cell infiltration and the expression of some types of immune checkpoints. Relevant functional analyses revealed a role for *GJB2* in extracellular matrix remodeling and activation of cancer-related signaling pathways. Eventually, through the single-cell sequenced data, we concluded that *GJB2* related hub-genes were involved in intercellular communication by influencing the SPP1 signaling pathway. One mechanism by which *GJB2* exerts its cancer-specific relevant effects could be traced back to the changes in this pathway. A promising strategy for LUAD research was provided.

## Data availability statement

The original contributions presented in the study are included in the article/**Supplementary Material**. Further inquiries can be directed to the corresponding authors.

## Author contributions

ZZ conceived the study, obtained funding. LZ designed study strategy. ZX acquired the data and drafted the manuscript. XW critically revised the manuscript. ZL performed statistical analysis and technical support. All authors contributed to the article and approved the submitted version.

## Supplementary material

Supplementary Figure 1 Supplementary The distribution and overlap of core genes of the first 25 enriched gene sets.

Supplementary Figure 2 Quality control of single cells in LUAD samples. **(A)** Relationship between the percentage of mitochondrial genes and mRNA reads or relationship between the number of mRNA and mRNA reads. **(B)** Scatterplot of the top 1500 highly variable genes. **(C)** Violin-plot before quality control illustrating the number of genes and percentage of mitochondrial genes in each cell type from nine samples. **(D)** Principal component analysis of each sample. **(E)** Significant principal components were identified using the jackStraw function. **(F)** Heat map of each principal component feature gene.

Supplementary Figure 3 A Heat map of the most important differentially expressed genes in different cell clusters. B. Marker genes for each cell type.

Supplementary Figure 4 Hub-genes expression in single-cell datasets. **(A)** Violin plots of hub-genes in single-cell datasets. **(B)** Expression of related genes in the SPP1 signaling pathway in different cell types.
