# Supplementary figures and images for: Ion channel gene GJB2 influences the intercellular communication by Up-regulating the SPP1 signaling pathway identified by the single-cell RNA sequencing in lung adenocarcinoma
Source: Front Oncol. 2023 Apr 28;13:1146976. doi: 10.3389/fonc.2023.1146976 (PMC10175797; doi:10.3389/fonc.2023.1146976)

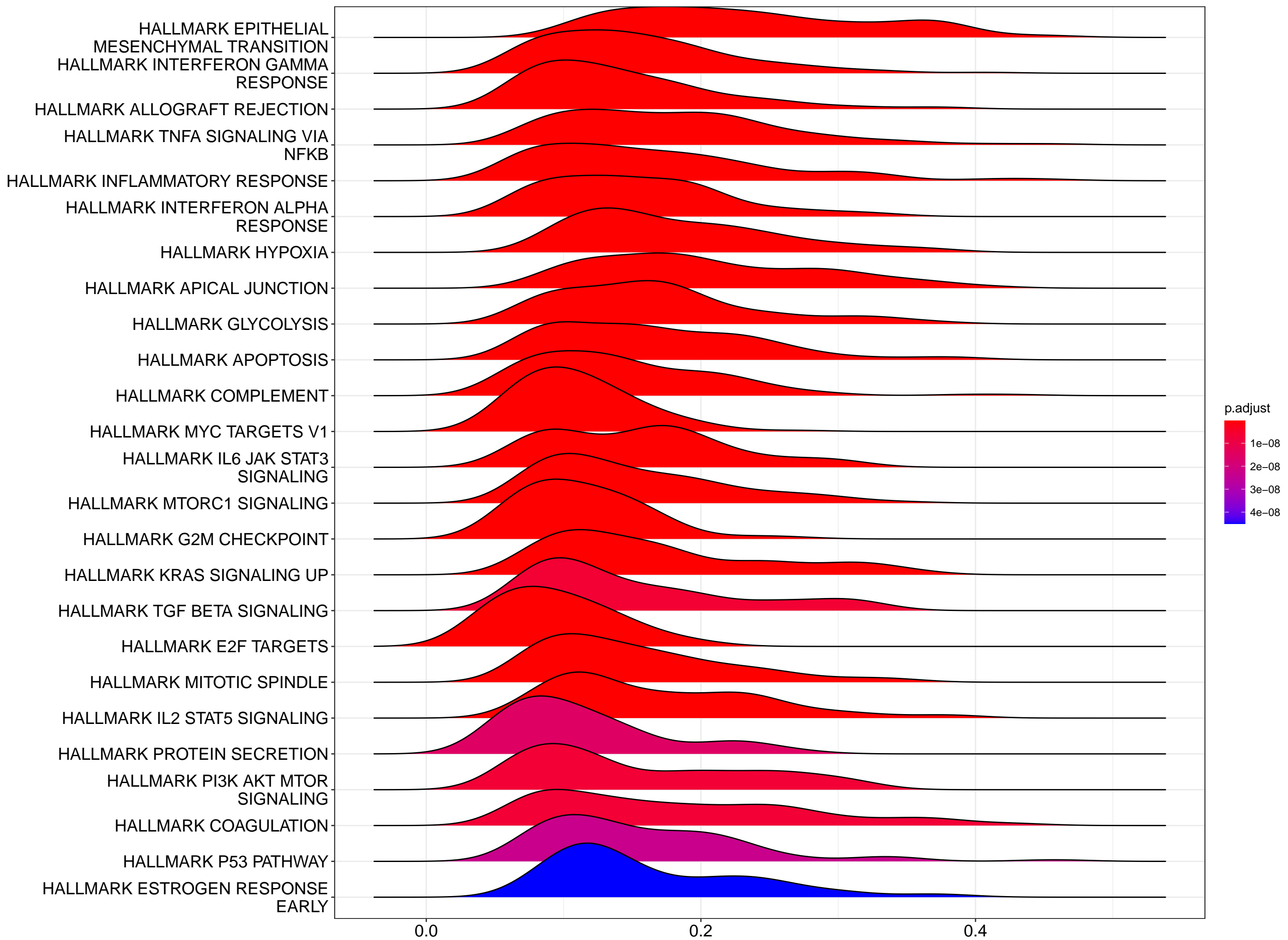

Supplement: Supplementary Figure 1 — Supplementary The distribution and overlap of core genes of the first 25 enriched gene sets. [file Image_1.pdf]

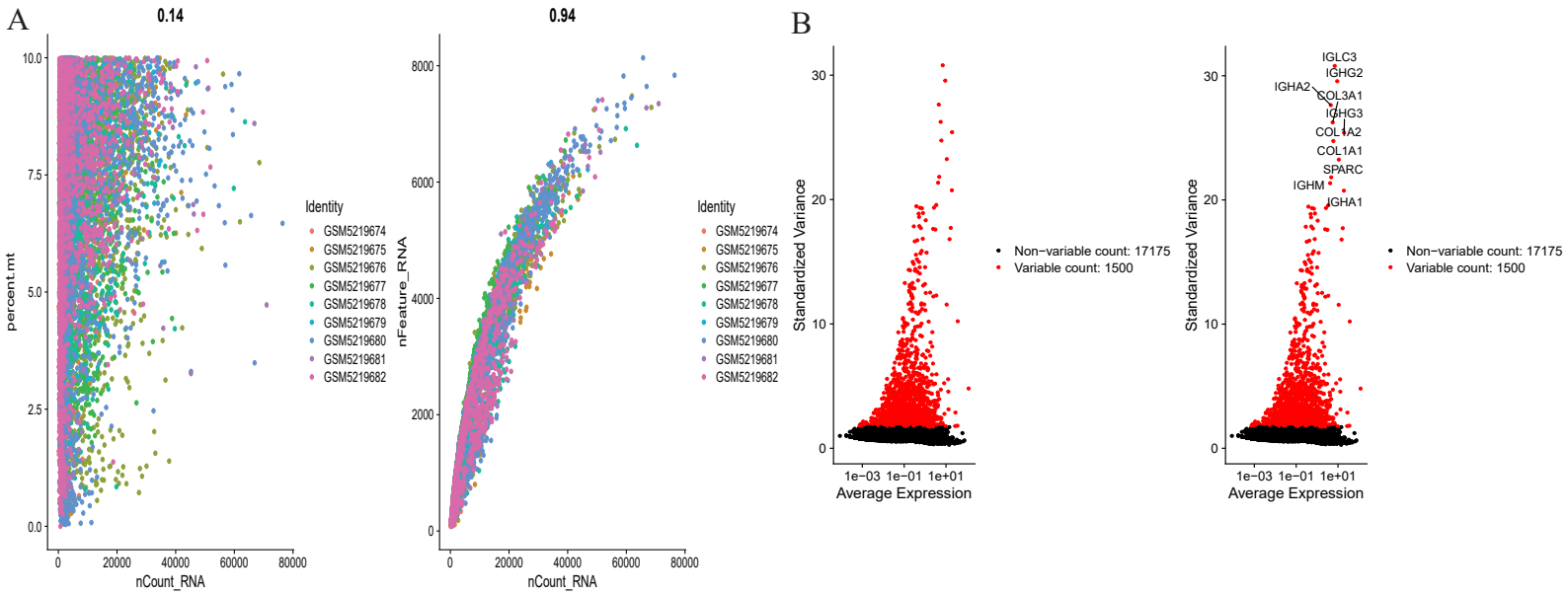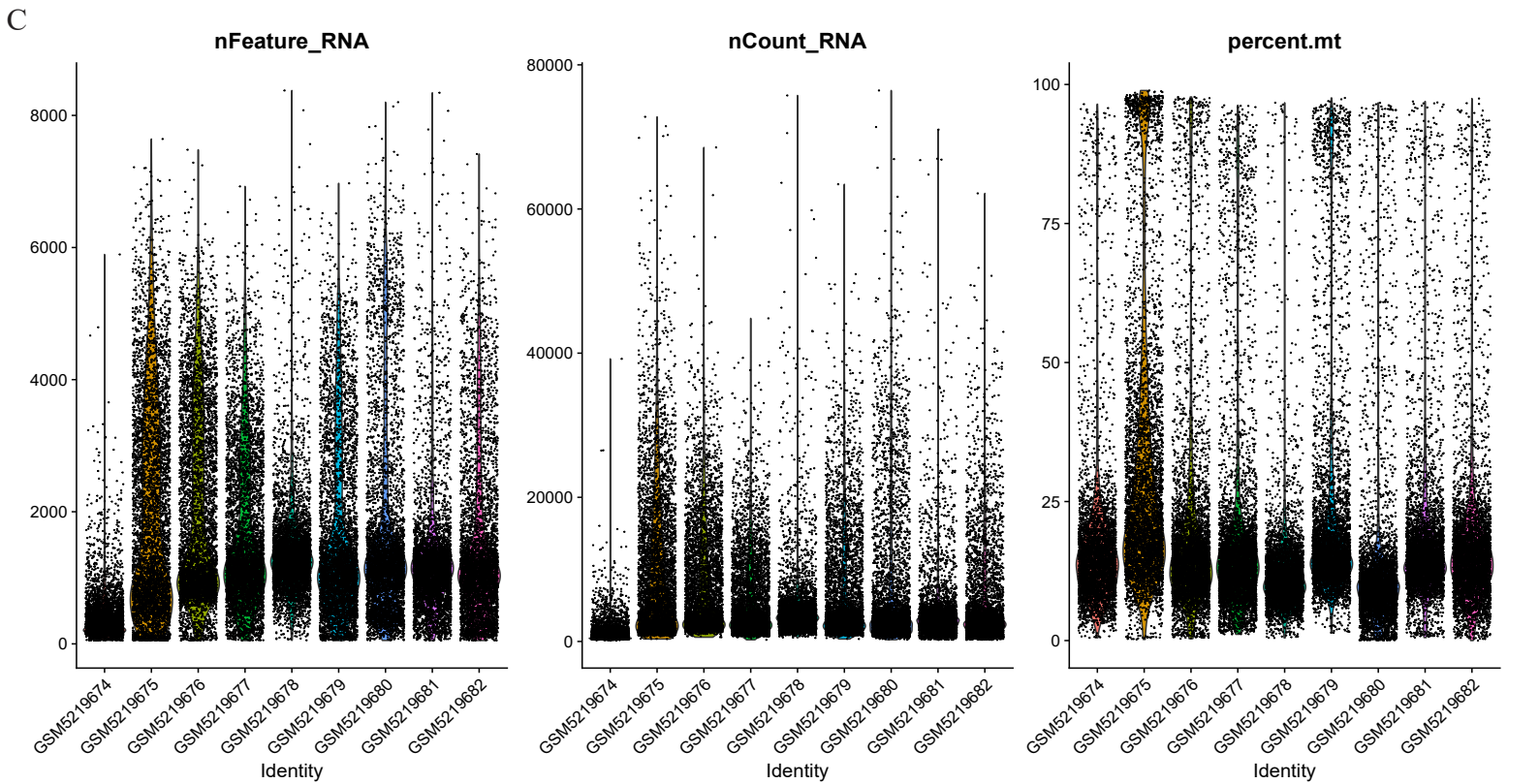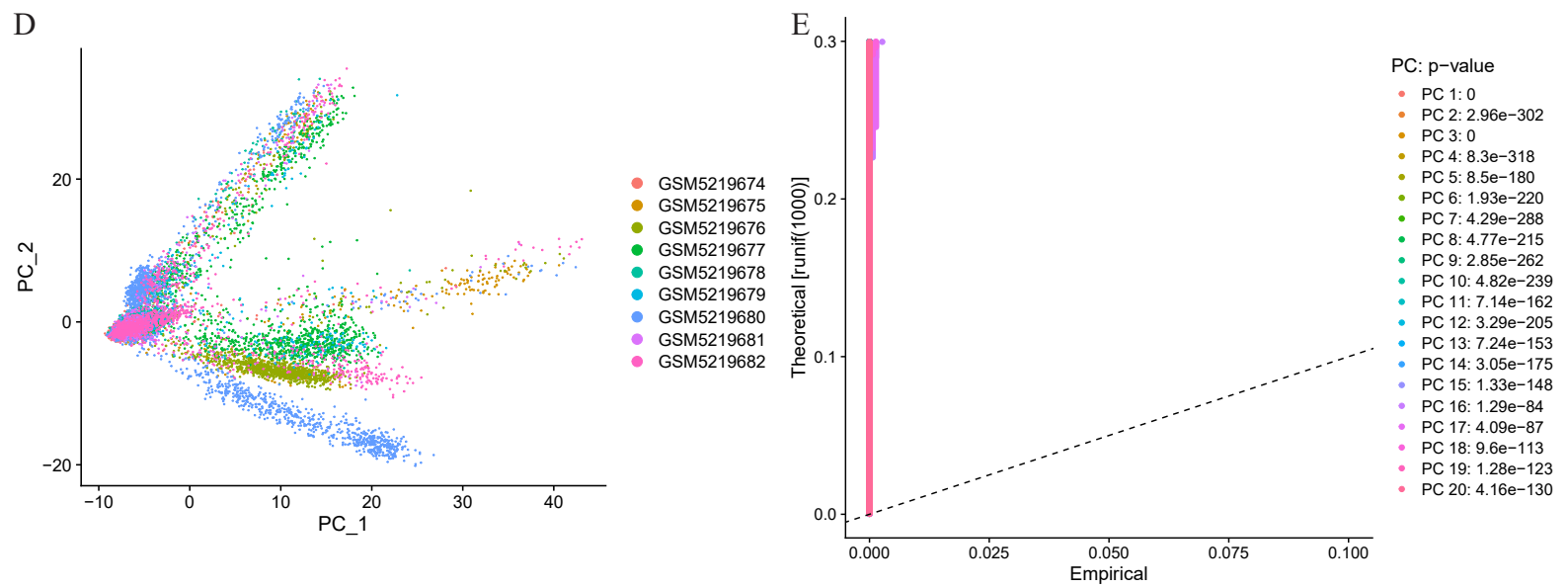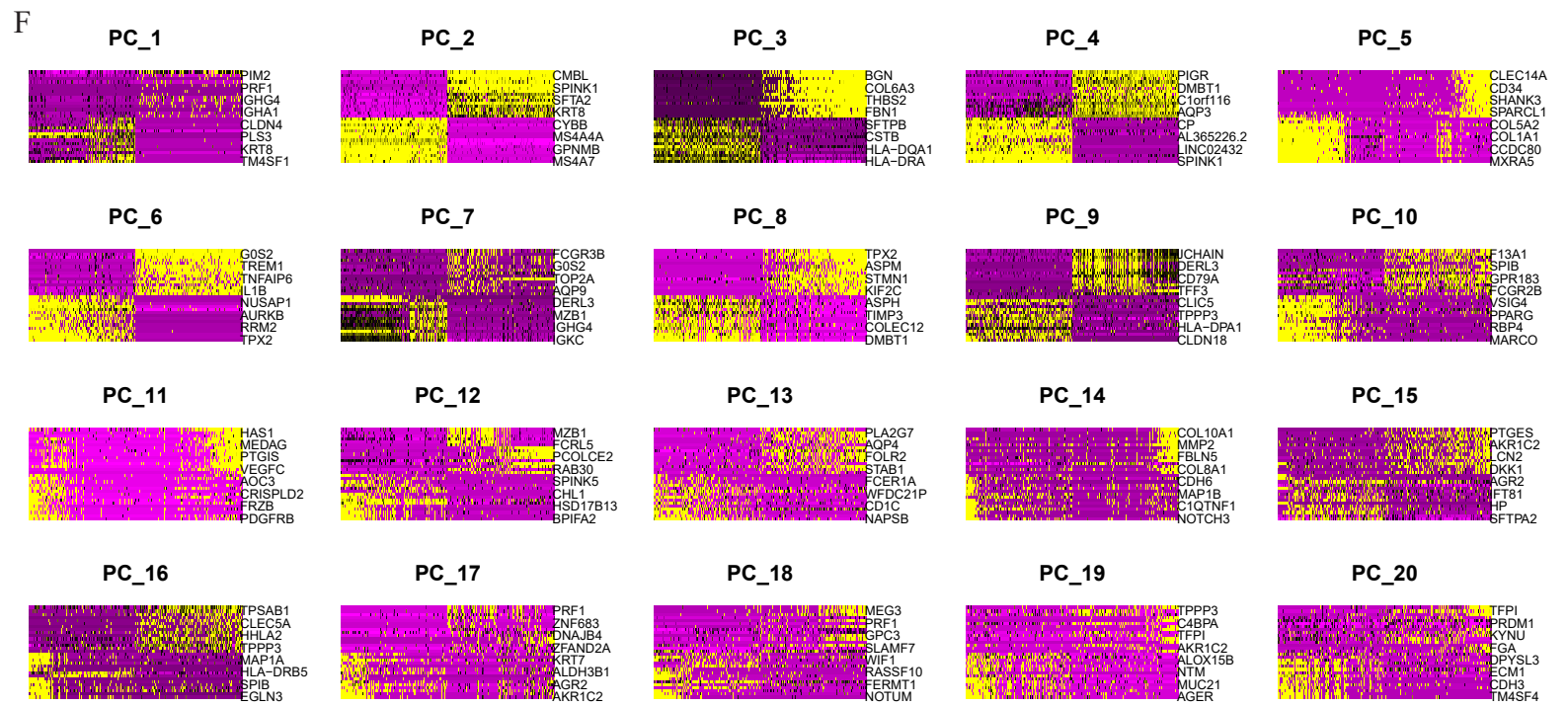

Supplement: Supplementary Figure 2 — Quality control of single cells in LUAD samples. (A) Relationship between the percentage of mitochondrial genes and mRNA reads or relationship between the number of mRNA and mRNA reads. (B) Scatterplot of the top 1500 highly variable genes. (C) Violin-plot before quality control illustrating the number of genes and percentage of mitochondrial genes in each cell type from nine samples. (D) Principal component analysis of each sample. (E) Significant principal components were identified using the jackStraw function. (F) Heat map of each principal component feature gene. [file Image_2.pdf]

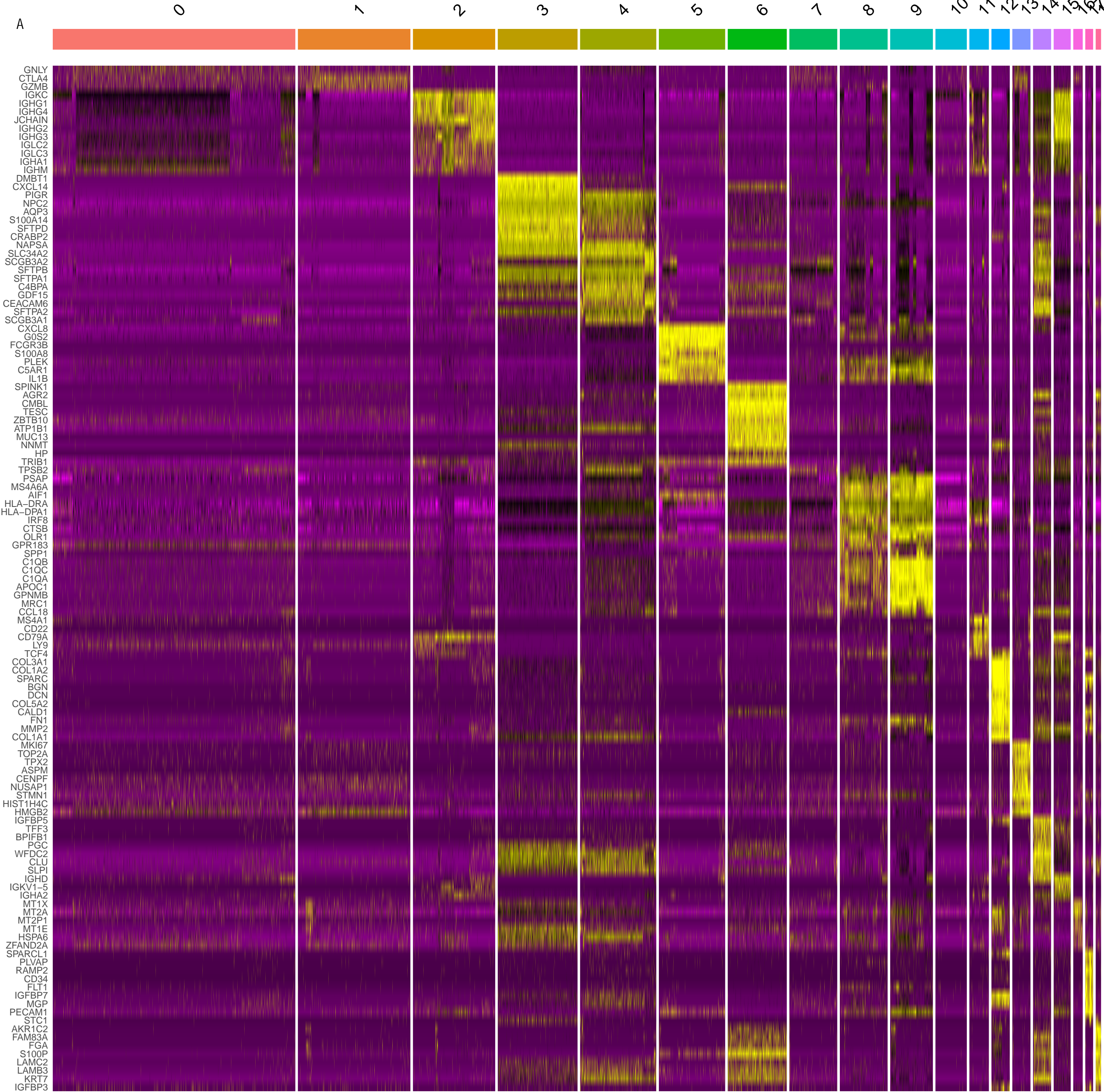

## Epithelial\_cells

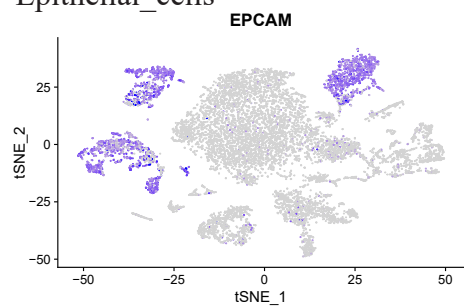

## Macrophage

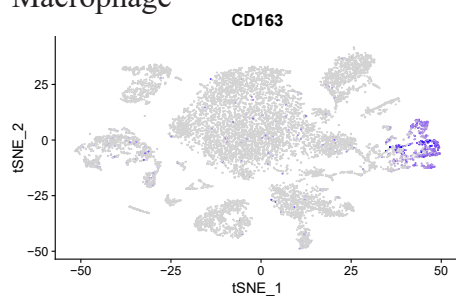

## Fibroblasts

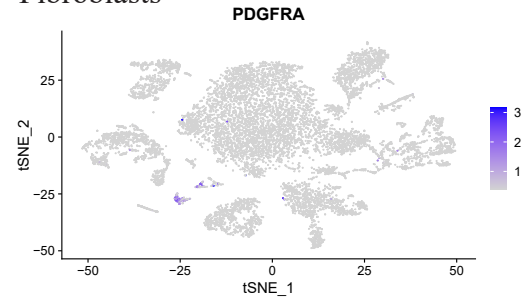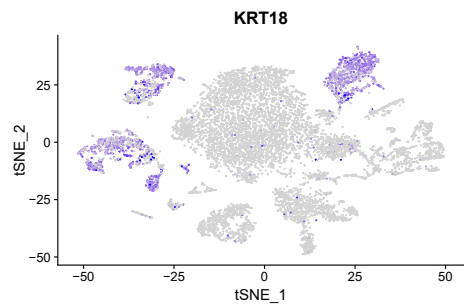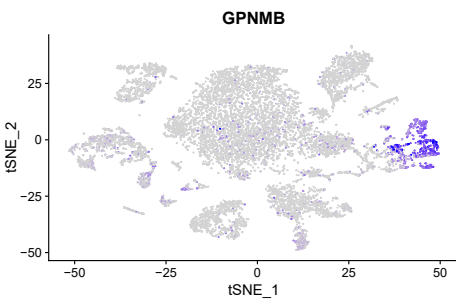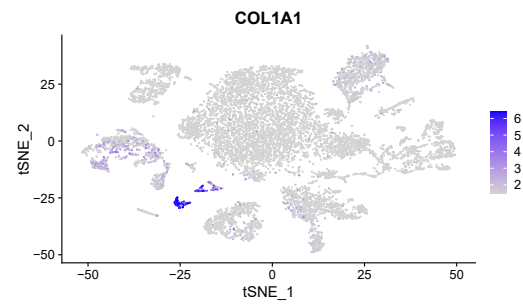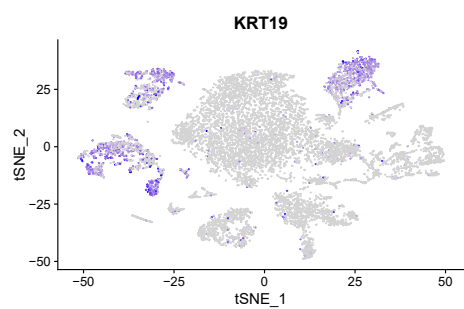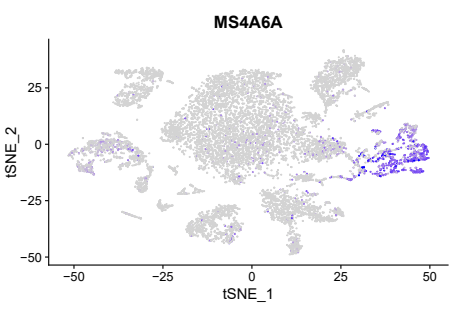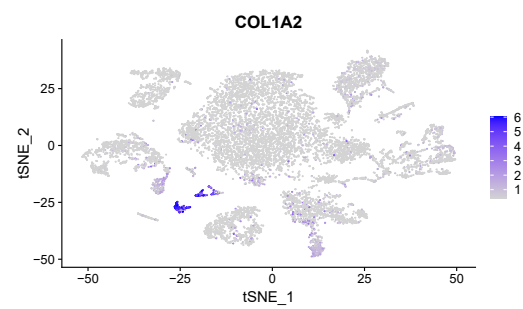

## T\_cells

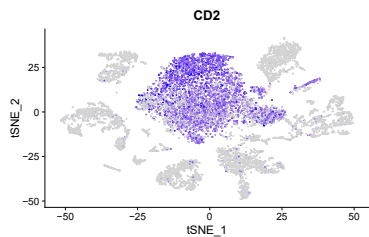

## B\_cells

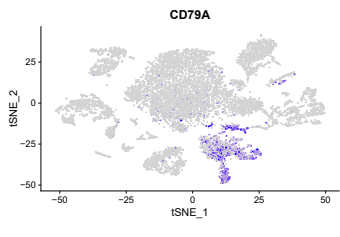

## Neutrophils

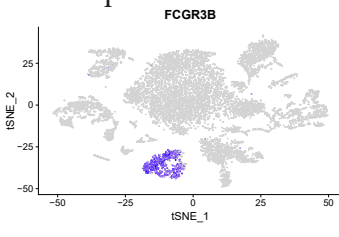

## Endothelial\_cells

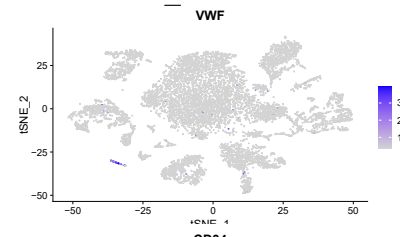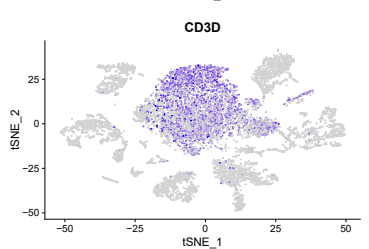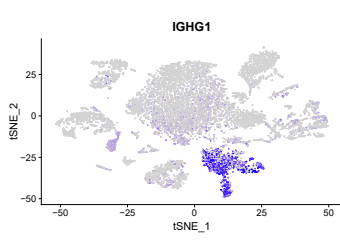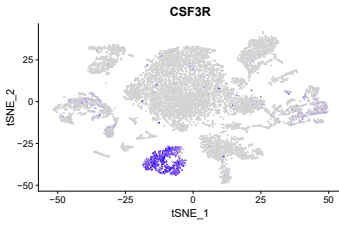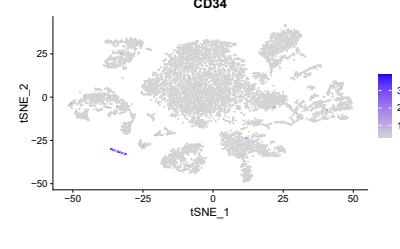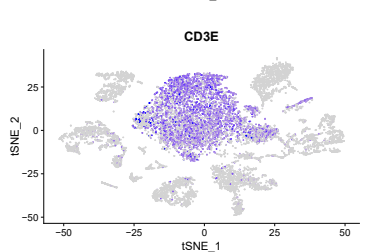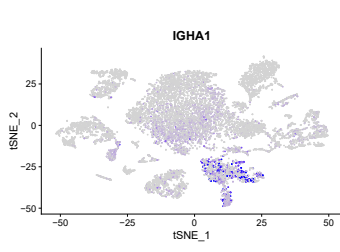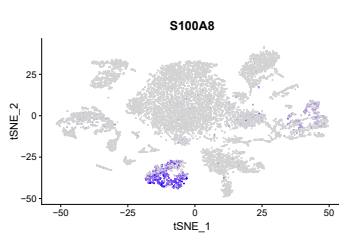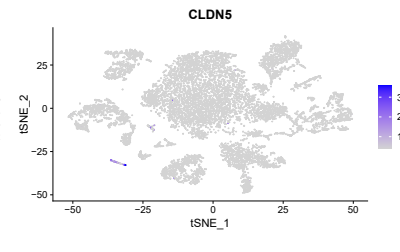

Supplement: Supplementary Figure 3 — A Heat map of the most important differentially expressed genes in different cell clusters. B. Marker genes for each cell type. [file Image_3.pdf]

A

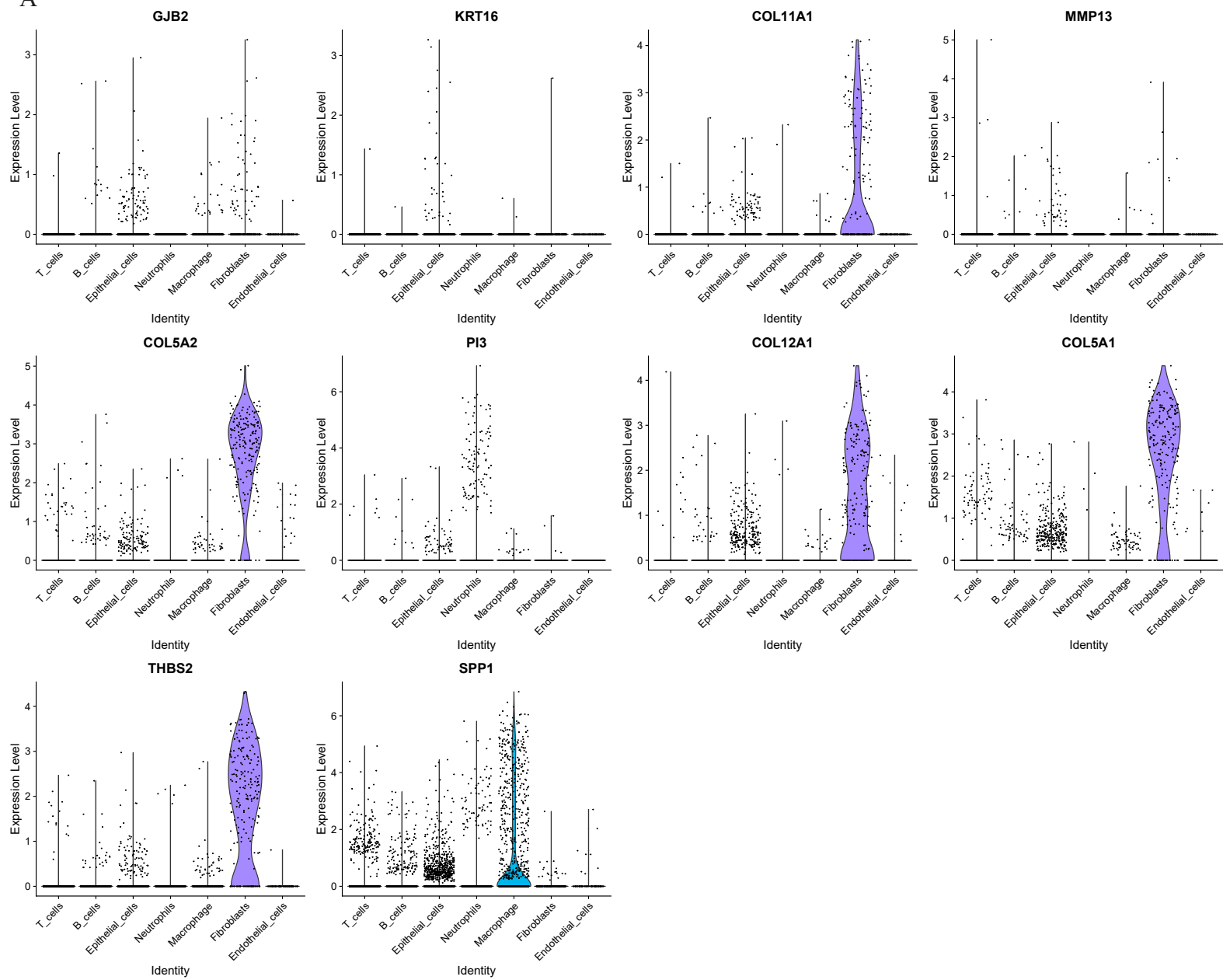

B

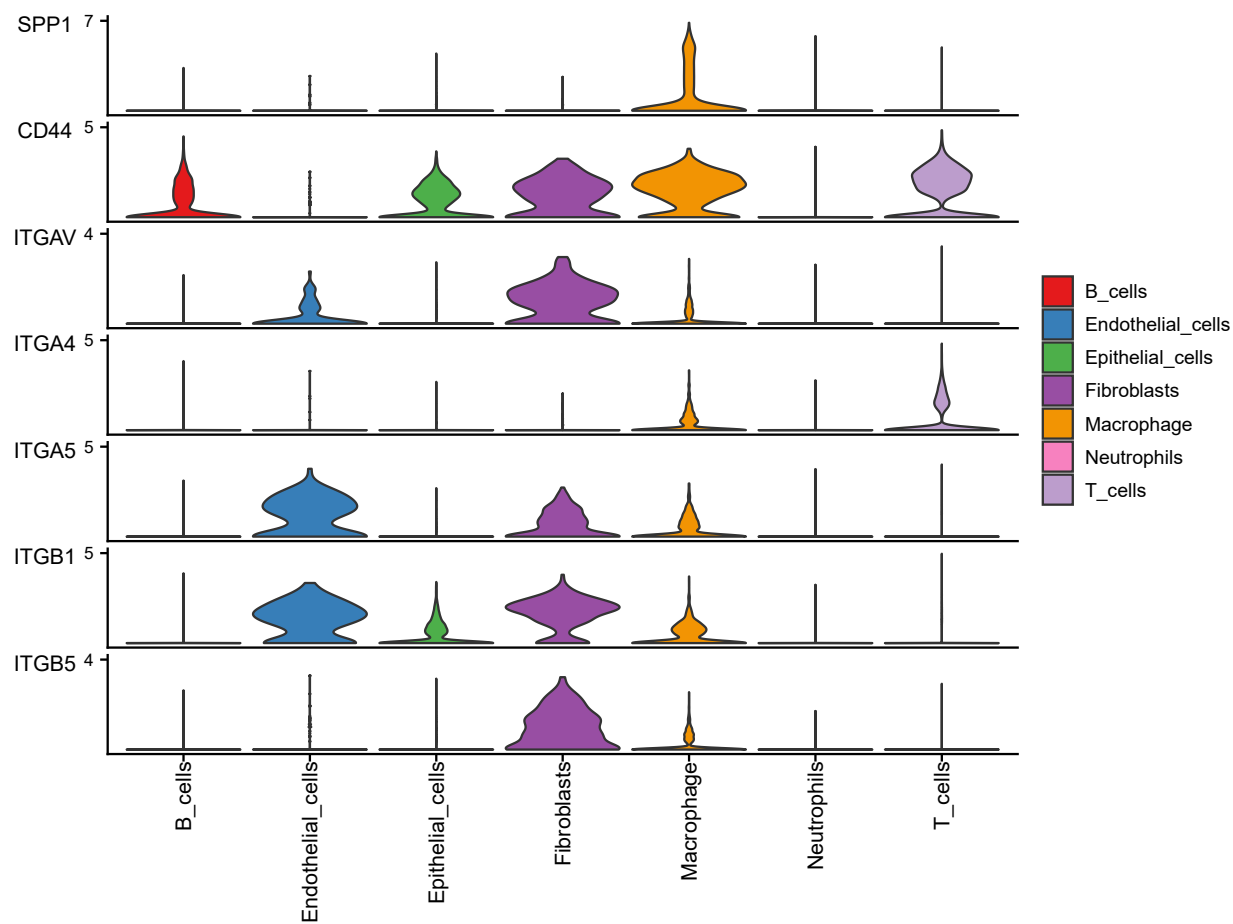

Supplement: Supplementary Figure 4 — Hub-genes expression in single-cell datasets. (A) Violin plots of hub-genes in single-cell datasets. (B) Expression of related genes in the SPP1 signaling pathway in different cell types. [file Image_4.pdf]

A

GJB2 high expression group

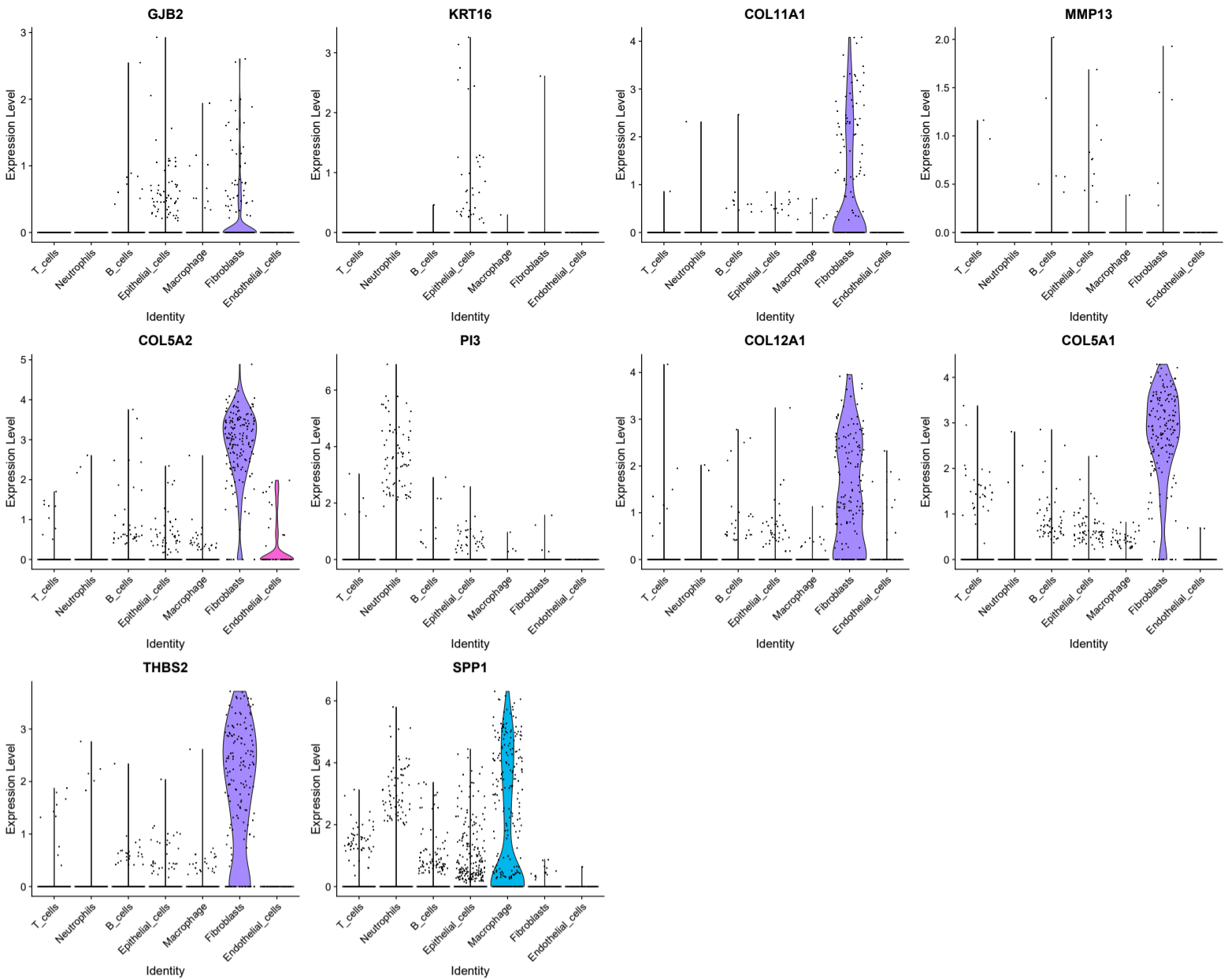

B

GJB2 low expression group

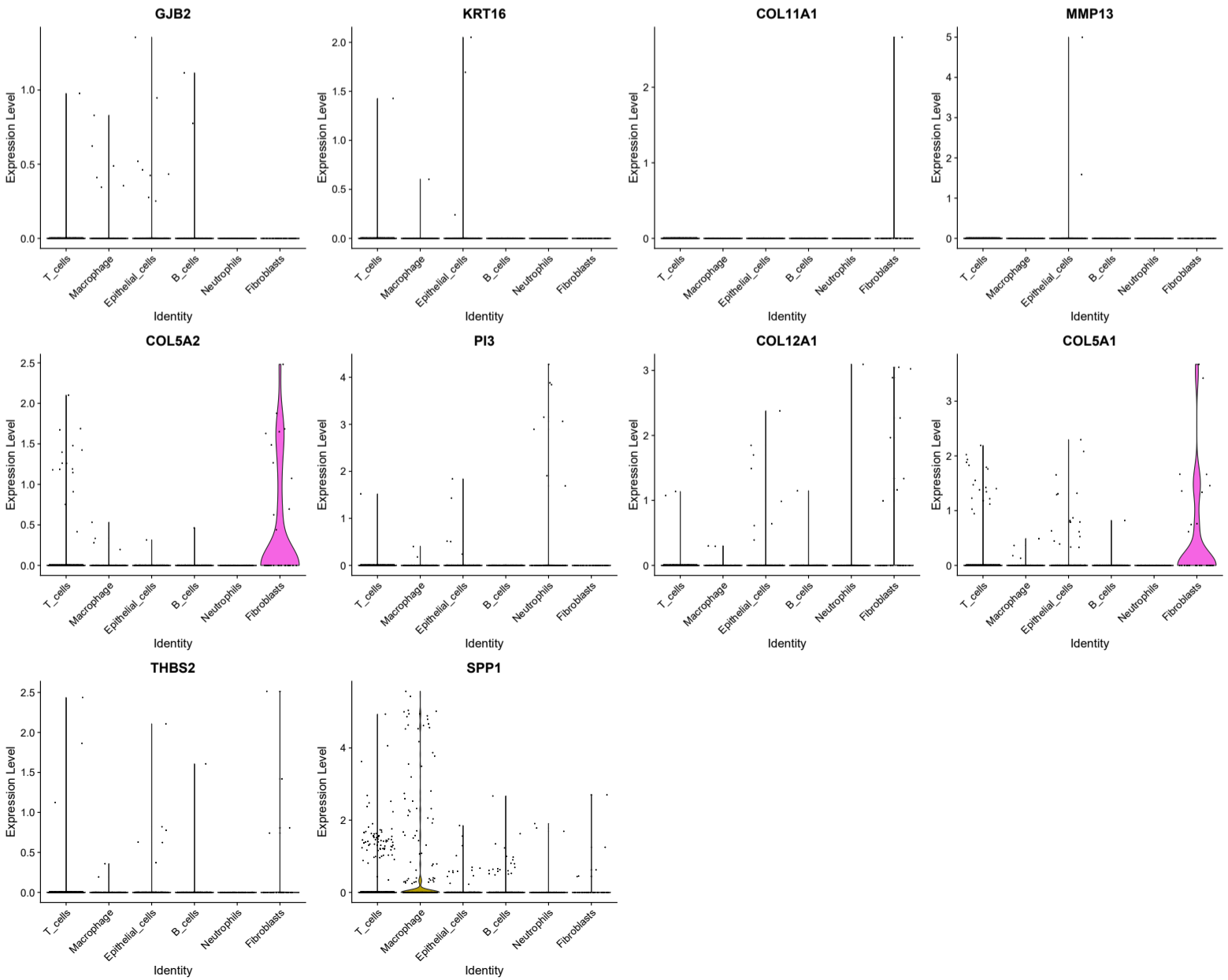

Supplement: Supplementary file 5 [file Image_5.pdf]
